# Association of Oral Anticoagulants and Verapamil or Diltiazem With Adverse Bleeding Events in Patients With Nonvalvular Atrial Fibrillation and Normal Kidney Function

**DOI:** 10.1001/jamanetworkopen.2020.3593

**Published:** 2020-04-24

**Authors:** Phuong Pham, Stephan Schmidt, Lawrence Lesko, Gregory Y. H. Lip, Joshua D. Brown

**Affiliations:** 1 Department of Pharmaceutical Outcomes & Policy, University of Florida College of Pharmacy, Gainesville; 2 Center for Pharmacometrics and Systems Pharmacology, University of Florida, Orlando; 3 Liverpool Centre for Cardiovascular Science, University of Liverpool and Liverpool Heart & Chest Hospital, Liverpool, United Kingdom; 4 Aalborg Thrombosis Research Unit, Department of Clinical Medicine, Aalborg University, Aalborg, Denmark; 5 Center for Drug Evaluation & Safety, University of Florida, Gainesville

## Abstract

**Importance:**

Direct oral anticoagulants (DOACs) are purported to have fewer drug-drug interactions than warfarin. However, potential interactions with coprescribed medications are still a safety concern. Verapamil hydrochloride and diltiazem hydrochloride are combined P-glycoprotein (P-gp) and CYP3A4 inhibitors and may be associated with increases in the risk of bleeding with DOACs.

**Objective:**

To evaluate the risk of bleeding with DOACs and verapamil or diltiazem using an active comparator design.

**Design, Setting, and Participants:**

A comparative effectiveness active comparator cohort study was conducted using US population-based data (2010-2015) analyzed between January 1 and July 15, 2019. Data were obtained on 48 442 patients with nonvalvular atrial fibrillation who had received an index prescription of dabigatran, rivaroxaban, or apixaban between October 19, 2010, through June 30, 2015, with final follow-up on October 1, 2015. Analysis was restricted to individuals with no history of kidney disease who were receiving standard doses of the DOACs.

**Exposures:**

Patients with initial prescriptions of DOACs who were receiving verapamil or diltiazem were compared with those receiving amlodipine or metoprolol.

**Main Outcomes and Measures:**

Overall and gastrointestinal major, moderate, and minor bleeding using primary or secondary diagnoses. Hazard ratios and 95% CIs were estimated using inverse probability of treatment weights in Cox proportional hazards regression models.

**Results:**

Of the 48 442 patients reviewed, analysis was conducted on 1764 patients receiving DOACs with verapamil or diltiazem compared with 3105 receiving amlodipine and 1793 patients receiving DOACs with verapamil or diltiazem compared with 3224 receiving metoprolol. Depending on the comparison, approximately 60% of the cohort were younger than 65 years and male, which differed by treatment group. Rivaroxaban and apixaban were not associated with increased rates of bleeding for patients receiving verapamil or diltiazem compared with those receiving amlodipine or metoprolol. Among patients receiving dabigatran etexilate, the overall bleeding rate was 52% higher (hazard ratio, 1.52; 95% CI, 1.05-2.20) with verapamil or diltiazem vs amlodipine and 43% higher (hazard ratio, 1.43; 95% CI, 1.02-2.00) vs metoprolol. Bleeding rates for dabigatran with verapamil or diltiazem were higher overall for other bleeding types (244.9 vs 158.4 per 1000 person-years; adjusted hazard ratios of overall GI bleeding: 2.16; 95% CI, 1.30-3.60; minor bleeding: 1.56; 95% CI, 1.07-2.27; and minor GI bleeding: 2.16; 95% CI, 1.29-3.63). Sensitivity analyses showed consistent results for dabigatran when used with verapamil and diltiazem, with magnitudes ranging from 50% to 100% increased hazard rates and no significant results for apixaban or rivaroxaban.

**Conclusions and Relevance:**

Current US prescribing information only recommends prescribing changes with dabigatran and P-gp inhibitors with lower kidney function. This study found increased bleeding risk associated with dabigatran when used concomitantly with the P-gp inhibitors verapamil and diltiazem in individuals with normal kidney function. Clinicians and patients may need to consider these drug-drug interactions when choosing oral anticoagulation.

## Key Points

**Question:**

What is the association of oral anticoagulants and verapamil hydrochloride or diltiazem hydrochloride with adverse bleeding events in patients with no kidney disease?

**Findings:**

In this comparative effectiveness study using data from 48 442 patients, rates of bleeding were increased for patients receiving dabigatran etexilate with concomitant verapamil or diltiazem compared with those who were receiving concomitant amlodipine or metoprolol therapy. Other direct oral anticoagulants had no evidence of these drug-drug interactions.

**Meaning:**

These findings suggest that prescribers may need to avoid P-glycoprotein–related drug-drug interactions with dabigatran regardless of kidney function.

## Teaser

This comparative effectiveness study evaluates drug-drug interactions between direct oral anticoagulants and verapamil or diltiazem in patients with no history of kidney disease.

## Introduction

Direct oral anticoagulants (DOACs), first introduced into the market in 2010, have become more popular as a stroke prevention therapy for patients with nonvalvular atrial fibrillation owing to less complexity in therapeutic dosing in addition to equivalent efficacy and superior safety compared with warfarin.^1^ Even though adverse bleeding events are of less concern among patients receiving DOACs compared with those receiving traditional warfarin therapy, the risk of major bleeding events is still present. This risk can be further increased when DOACs are administered with other medications that inhibit their metabolic or absorption pathways, such as through CYP3A4 and P-glycoprotein (P-gp) inhibition.^2,3^

The prevalence of hypertension is high among patients with nonvalvular atrial fibrillation—about 70% to 90% based on data from randomized clinical trials of DOACs^4,5,6^—and use of antihypertensive drugs is common. Verapamil hydrochloride and diltiazem hydrochloride are nondihydropyridine calcium-channel blocking antihypertensives that are also recommended as a heart rate control therapy for patients with nonvalvular atrial fibrillation.^7,8^ As both drugs are combined P-gp and CYP3A4 inhibitors, they can contribute to clinically relevant drug-drug interactions when coadministered with DOACs.^3^ Little direct clinical evidence has been provided regarding these drug-drug interactions. An observational study by Chang et al^9^ found no increased bleeding risk among patients receiving both DOACs and verapamil or diltiazem compared with those receiving DOACs alone. However, the lack of active comparators in that study could lead to insurmountable biases and residual confounding as pointed out by several response letters.^10,11,12,13,14^ Suspected drug-drug interactions should be evaluated individually using active control groups strategically selected to better overlap either in biological target or therapeutic use to the exposure of interest.^15,16^

Our objective was to conduct a targeted comparative safety analysis of the potential drug-drug interaction between verapamil or diltiazem and DOACs using an active comparator study design to reduce the residual confounding present in existing real-world studies. An ideal active comparator should be either of the same medication class or be used for similar purposes as the reference product. Therefore, we selected 2 different comparators: amlodipine, a dihydropyridine calcium-channel blocking antihypertensive drug, and metoprolol, a β-blocker antihypertensive that is also recommended for heart rate control in patients with nonvalvular atrial fibrillation.^17^ These 2 drugs are not classified as P-gp or CYP3A4 inhibitors and provide similar pharmacologic pathways and therapeutic uses compared with verapamil and diltiazem.^18^ We aimed to compare the risk of major, moderate, and minor adverse bleeding events among patients with concomitant use of DOACs and verapamil or diltiazem vs concomitant use of DOACs and amlodipine or metoprolol.

## Methods

### Study Population

This was a retrospective comparative effectiveness cohort study using IBM Watson MarketScan Databases, including the Commercial Claims and Medicare Supplemental Database, which represent the health care use and encounter data for 20 to 40 million individuals annually in the US. The data for this study were obtained from enrollment files, medical inpatient and outpatient claims, and pharmacy claims. Data were analyzed between January 1 and July 15, 2019. Use of the data was considered exempt from human subjects review by the University of Florida Institutional Review Board. This study followed the International Society for Pharmacoeconomics and Outcomes Research (ISPOR) reporting guideline for comparative effectiveness studies.

All patients receiving DOACs (dabigatran, rivaroxaban, and apixaban) were identified from October 19, 2010, through June 30, 2015. The date of the first prescription filled of each medication was defined as the index date. Patients were required to have at least 1 inpatient or 2 outpatient diagnoses of nonvalvular atrial fibrillation within 60 days before the index date based on *International Classification of Diseases, Ninth Revision*, code 427.31. Patients were included if they had at least 12 months of continuous enrollment within the Commercial Claims and Medicare Supplemental Database before the index date and excluded if they had received a prescription for any of the study medications (DOACs or antihypertensives) during the baseline period to ensure an anticoagulation-naive cohort. We also excluded patients with more than 1 DOAC dispensed on the index date, as well as those with a diagnosis of mitral valve disease, heart valve repair or replacement, or joint replacement during the baseline period consistent with exclusion criteria in clinical trials and current indications for DOACs.

Owing to the potential dose adjustment and inherent selection bias associated with using DOACs with verapamil or diltiazem, we further restricted the sample to patients with normal kidney function and standard DOAC doses. Normal kidney function was defined as not having any type of acute or chronic kidney disease or receiving dialysis during the 1-year preindex period. The usual drug doses were considered to be 150 mg twice daily for dabigatran, 20 mg once daily for rivaroxaban, and 5 mg twice daily for apixaban, per current US prescribing information labels.^19,20,21^ All diagnosis and procedural codes used for baseline demographics, inclusion, and exclusion criteria are included in eTables 1-3 in the Supplement.

### Design

Our exposure of interest was the concomitant use of DOACs and verapamil or diltiazem. The comparison group was DOACs with concomitant use of amlodipine or metoprolol. Use of verapamil or diltiazem, amlodipine, and metoprolol were identified within the 6 months before DOAC therapy initiation. Patients were considered exposed to one of these drugs if they had an overlap use with DOACs on the index date and at least 90 days of cumulative use of these drugs within the 6-month preindex period. Prescription fills of all studied medications were identified based on the National Drug Codes. Medication use each day during follow-up was assessed using the prescription fill dates and days-supplied values on prescription claims with correction for overlapping refills.

Baseline characteristics, including patient demographic data (age, sex, and region), medical history (diabetes, hypertension, hyperlipidemia, heart failure, stroke, bleeding, liver disease, dementia, vascular disease, chronic pulmonary disease, rheumatic disease, cancer, metastatic cancer, smoking, Charlson Comorbidity Index burden,^38^ CHA_2_DS_2_-VASc [congestive heart failure, hypertension, age ≥75 years (doubled), diabetes, stroke/transient ischemic attack/thromboembolism (doubled), vascular disease (prior myocardial infarction, peripheral artery disease, or aortic plaque), age 65-75 years, sex category (female)] score,^22^ and HAS-BLED [hypertension, abnormal renal and liver function, stroke, bleeding, labile international normalized ratio, elderly, and drugs or alcohol] Score^23^), other medication use, and health care use (outpatient visit, number of medications used, and plan type) were assessed during the 1-year preindex period. The Charlson Comorbidity Index includes 17 comorbidities, with higher numbers indicating higher comorbidity burden; CHA_2_DS_2_-VASc is a risk score for stroke risk in nonvalvular atrial fibrillation that ranges between 0 and 9, with higher scores indicating higher stroke risk; and HAS-BLED is a risk score for major bleeding while the patient is receiving anticoagulants that ranges from 0 to 9, with higher scores indicating higher major bleed risk.

We used inverse probability of treatment weighting (IPTW) based on propensity scores to adjust for confounding.^24^ A propensity score was calculated based on all baseline covariates in logistic regression models that predicted the probability of receiving a specific treatment given all measured baseline factors. Separate models stratified by the DOAC prescribed and estimated the probabilities of receiving verapamil or diltiazem vs amlodipine and the probability of receiving verapamil or diltiazem vs metoprolol. Each patient was assigned a weight that was equal to the inverse of the predicted probability. These weights were corrected for extreme values by capping weights at a maximum of 10. Theoretically, once weights were applied, the treatment groups should be balanced on all measured baseline covariates providing a pseudorandomized framework that mitigates selection bias in the sample. Standardized differences were used to evaluate the effectiveness of these propensity score techniques, with a standardized difference less than 10% being considered sufficient balance.^25^

Follow-up began from the index date until when patients had a 7-day gap in any of the medication use (DOACs or antihypertensives), switched to any another study medication (ie, a different DOAC or different comparator), end of health care enrollment, or end of study period on October 1, 2015. A 7-day gap was chosen to make sure that patients were always receiving an anticoagulant given the short half-life of DOACs.^19,20,21^ Reentry into the cohort was not allowed if a medication was reinitiated after these events.

Study outcomes included major bleeding, moderate bleeding, and minor bleeding, which were identified by published, validated *International Classification of Diseases, Ninth Revision, Clinical Modification*, diagnosis codes (eTable 4 in the Supplement).^26^ To reduce false-positive findings, we only considered the first and second diagnosis or procedural code positions in the claim records. Major bleeding was defined as bleeding requiring hospitalization and meeting 1 of these criteria: (1) occurred at a critical site, (2) required a transfusion, or (3) led to death as described in Cunningham et al^26^ (eTable 4 in the Supplement). Moderate bleeding was considered bleeding events in the inpatient setting or emergency department that did not meet the criteria for major bleeding. Minor bleeding was defined as any bleeding treated on an outpatient basis. In our analysis, we combined major and moderate bleeding since major bleeding was rare. We also separated gastrointestinal (GI) bleeding for each category and analyzed these outcomes in separate analyses.

### Statistical Analysis

Baseline characteristics during the preindex period are reported as proportions for categorical variables and means with SDs for continuous variables. A Cox proportional hazards regression model was used to estimate hazard ratios (HRs) and 95% CIs for each outcome. All regression analyses were conducted on the weighted cohorts with stabilized IPTW to minimize potential confounding by measured baseline characteristics. Separate models were estimated for the comparison of patients receiving verapamil or diltiazem vs amlodipine or metoprolol for each DOAC and each bleed outcome. All analyses were performed with SAS, version 9.4 (SAS Institute Inc) and, using 2-tailed testing, the statistical significance level was set at *P* = .05. Study design and methods are reported consistently with updated recommendations for reproducibility and validity assessment.^27^

## Results

**Figure 1.**
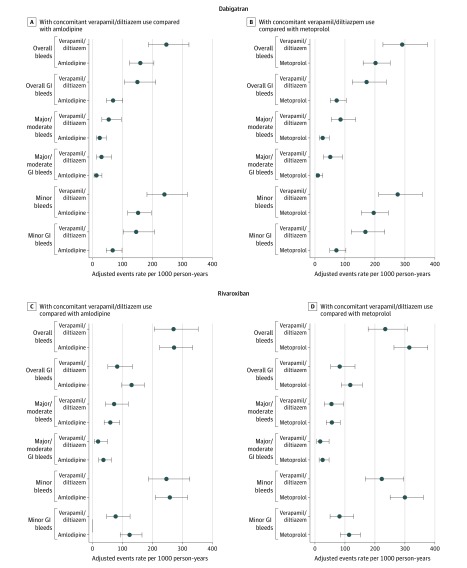
Adjusted Event Rates Comparing Verapamil or Diltiazem vs Amlodipine and Metoprolol Users Separately Stratified by Oral Anticoagulant (Dabigatran and Rivaroxaban) and Bleeding Site Error bars indicate 95% CIs. GI indicates gastrointestinal.

**Figure 2.**
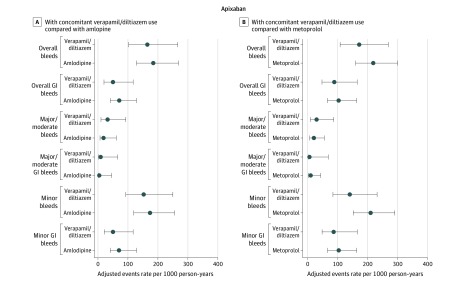
Adjusted Event Rates Comparing Verapamil or Diltiazem vs Amlodipine and Metoprolol Users Separately Stratified by Oral Anticoagulant (Apixaban) and Bleeding Site Error bars indicate 95% CIs. GI indicates gastrointestinal.

There were a total of 48 442 patients receiving new DOAC therapy with a diagnosis of nonvalvular atrial fibrillation identified during the study period from 2010 to 2015 (eFigure 1 in the Supplement). After consideration of the inclusion and exclusion criteria, the cohorts included 1764 patients receiving DOACs with verapamil or diltiazem compared with 3105 patients receiving DOACs with amlodipine. Adjusted event rates and 95% CIs are shown in Figure 1 and Figure 2. There were 1793 patients receiving DOACs with verapamil or diltiazem compared with 3224 patients receiving DOACs with metoprolol. These cohort numbers differed slightly due to exclusion of prior medication users. Among all patients, DOAC use overall was approximately 40% for both dabigatran etexilate and rivaroxaban and 20% for apixaban, which was similarly distributed among all treatment groups. Characteristics differed based on the cohort. Most (approximately 60%) of the cohorts identified were younger than 65 years and male depending on the specific comparison. Comorbidities were prevalent in the population, including diabetes (>25%), hypertension (80%), hypercholesterolemia (>60%), and heart disease (>25%). Approximately 40% of the cohort had a CHA_2_DS_2_-VASc score of 4 or greater and 70% to 75% had a HAS-BLED score between 0 and 2.

The verapamil or diltiazem vs amlodipine cohorts differed slightly in baseline characteristics (Table 1). However, after IPTW, all baseline characteristics were balanced with standardized differences less than 10% (eTable 5 in the Supplement). The number of events and crude event rates are reported in eTable 7 in the Supplement. Patients receiving rivaroxaban (274.8 vs 270.1 per 1000 person-years) and apixaban (159.1 vs 207.7 per 1000 person-years) had similar bleeding rates regardless of verapamil, diltiazem, or amlodipine exposure. For dabigatran, however, there were statistically significant increases in overall bleeding rates (244.9 vs 158.4 per 1000 person-years) and in the adjusted HRs of overall bleeding (HR, 1.52; 95% CI, 1.05-2.20), overall GI bleeding (HR, 2.16; 95% CI, 1.30-3.60), minor bleeding (HR, 1.56; 95% CI, 1.07-2.27), and minor GI bleeding (HR, 2.16; 95% CI, 1.29-3.63). Combined bleeding categories included large point estimates for major/moderate bleeding (HR, 2.27; 95% CI, 0.97-5.29) and major/moderate GI bleeding (HR, 2.27; 95% CI, 0.72-7.11), but the 95% CIs included the null (Table 2).

**Table 1.** Baseline Characteristic of Cohort of Direct Oral Anticoagulants With Verapamil or Diltiazem and With Amlodipine Use

Characteristic	Dabigatran		Rivaroxaban		Apixaban	
	Verapamil or diltiazem (n = 750)	Amlodipine (n = 1316)	Verapamil or diltiazem (n = 666)	Amlodipine (n = 1241)	Verapamil or diltiazem (n = 348)	Amlodipine (n = 548)
**Demographic, %**						
Age, y						
<65	56.93	49.16	63.51	52.86	56.32	49.27
65 to <70	12.53	13.07	11.71	14.67	15.52	15.69
70 to <75	11.07	15.43	9.46	13.94	12.07	17.88
75 to <80	10.93	12.23	10.36	11.6	11.21	10.40
80 to <85	6.80	7.45	3.60	5.32	3.45	4.93
≥85	1.73	2.66	1.35	1.61	1.44	1.82
Sex						
Women	45.87	36.78	45.65	35.29	46.55	35.95
Men	54.13	63.22	54.35	64.71	53.45	64.05
**Medical history, %**						
Diabetes without complication	26.27	32.07	23.27	32.8	30.17	36.50
Diabetes with complication	4.80	6.16	4.50	7.01	5.17	10.04
Hypertension	79.07	90.35	79.43	92.67	83.91	95.99
Hypercholesterolemia	54.13	62.01	58.71	67.04	64.66	71.35
Liver failure						
Mild	3.07	2.13	2.85	3.87	4.89	3.10
Moderate or severe	0.00	0.00	0.00	0.08	0.29	0.00
Cancer						
Solid tumors	15.20	15.2	15.77	15.63	18.39	18.8
Leukemias/lymphomas	1.60	1.52	1.35	1.61	1.44	0.18
Metastatic disease	1.20	0.84	1.05	0.97	1.15	1.64
Obesity	12.53	13.15	13.96	16.04	17.24	18.43
Peptic ulcer disease	0.67	0.38	0.75	0.64	0.00	0.55
Prior hospitalization for bleeding	8.67	7.14	8.26	7.41	12.36	8.03
Smoking	8.80	8.59	11.56	8.38	10.92	9.67
Cardiovascular disease						
Acute myocardial infarction						
Past 1-30 d	2.93	3.12	3.00	3.06	1.44	4.93
Past 31-180 d	1.73	1.98	1.95	1.29	3.45	2.37
Past 181-365 d	1.87	1.37	1.80	1.37	1.15	1.64
Coronary revascularization	8.00	8.89	6.16	7.98	10.34	7.12
Heart failure						
Hospitalized	9.87	9.50	7.66	11.28	7.76	10.04
Outpatient	11.73	11.32	13.36	13.94	10.63	14.60
Coronary artery disease	28.40	33.51	26.43	35.54	33.33	34.67
Other ischemic heart disease	25.87	30.78	23.42	33.36	30.75	32.66
Stroke						
Past 1-30 d	2.40	1.82	2.55	2.18	2.59	3.28
Past 31-180 d	1.47	1.98	1.65	1.77	3.16	1.82
Past 181-365 d	0.93	1.22	1.05	1.37	0.86	1.09
Other cerebrovascular disease	2.80	4.33	3.75	3.71	3.74	3.65
Transient ischemic attack	2.67	4.18	2.70	3.71	4.60	3.83
Cardioversion	14.93	19.07	13.36	18.45	20.4	22.63
Falls	1.33	0.99	1.95	2.01	2.59	3.47
Fractures	4.26	3.72	4.8	4.99	6.02	4.37
Syncope	5.60	6.53	7.66	8.94	7.76	7.85
Walker use	1.20	2.20	2.10	2.50	2.87	2.19
Charlson Comorbidity Index score, mean (SD)^a^	1.50 (1.78)	1.55 (1.67)	1.46 (1.74)	1.66 (1.70)	1.72 (1.89)	1.87 (1.92)
CHA_2_DS_2_-VASc score ^b^						
0-1	21.20	14.44	23.87	13.30	17.82	10.04
2	20.40	19.53	21.92	19.90	18.97	19.89
3	18.27	19.30	19.37	21.19	21.84	18.43
≥4	40.13	46.73	34.83	45.61	41.38	51.64
HAS-BLED score^c^						
0-1	36.80	28.04	39.94	24.74	27.87	21.17
2	38.80	45.29	35.74	43.76	41.38	45.8
3	19.33	20.44	19.97	24.82	22.70	25.36
≥4	5.07	6.23	4.35	6.69	8.05	7.66
**Medication use, %**						
General						
Estrogen therapy	6.40	4.71	4.50	3.30	4.31	4.38
Histamine_2_ antagonists	3.33	3.19	4.20	4.19	4.89	4.56
NSAID	20.40	24.01	22.82	25.79	27.01	24.45
Proton pump inhibitors	26.00	23.48	29.13	26.67	26.44	25.91
SSRI antidepressants	14.00	11.25	13.66	11.04	11.78	12.04
Cardiovascular						
ACE inhibitors	29.2	34.88	27.48	33.92	34.77	34.67
ARBs	20.93	28.42	22.22	29.01	20.98	30.47
Antiarrhythmics	33.73	22.95	41.44	23.37	37.07	24.45
Other anticoagulants	2.40	0.76	0.75	0.56	0.57	0.36
Antiplatelet	9.73	14.29	10.66	13.94	9.48	15.15
β-blocker	49.07	81.69	48.35	84.29	48.85	81.93
Digoxin	16.13	10.56	11.71	9.11	16.95	7.48
Diuretics						
Loop	17.60	19.76	15.62	19.50	18.10	17.15
Potassium sparing	7.33	9.42	6.76	8.14	7.76	8.94
Thiazide and other	31.20	42.63	29.28	44.16	33.91	41.06
Nitrates	5.07	8.43	7.06	7.74	6.61	8.58
Statins	52.00	63.53	47.75	62.45	54.31	63.50
Fibrates	3.87	6.99	4.65	5.80	5.17	4.74
Other antihyperlipidemic	8.80	12.01	7.21	8.86	6.61	9.31
Diabetes related						
Insulin	3.47	5.55	3.45	7.57	5.75	7.66
Metformin	15.33	20.21	13.66	19.58	18.97	22.45
Sulfonylurea	7.07	9.57	6.46	9.11	8.33	11.31
Other	8.67	11.25	7.36	10.56	8.91	12.96
Metabolic inhibitors						
Amiodarone	7.07	8.43	9.91	9.59	8.91	11.31
Dronedarone	9.60	7.83	10.81	5.00	8.62	6.02
Azole antifungals	17.60	15.96	18.47	17.89	19.54	14.96
Metabolic inducers						
Carbamazepine	0.27	0.08	0.30	0.40	0.00	0.36
Phenytoin	0.40	0.00	0.15	0.32	0.29	0.00
Phenobarbital	0.13	0.08	0.15	0.08	0.00	0.00
**Prescriber specialty**						
Internal medicine	11.47	14.06	11.56	14.67	10.34	11.86
Family practice	9.73	11.02	7.81	12.09	12.93	11.31
Geriatric medicine	0.00	0.08	0.00	0.08	0.00	0.00
Cardiology	40.13	41.26	41.44	37.79	35.06	39.78
Critical care medicine	0.00	0.08	0.30	0.00	0.00	0.18
Hematology	0.27	0.15	0.15	0.00	0.00	0.00
Oncology	0.27	0.00	0.15	0.08	0.00	0.00
Health care use						
Outpatient visits, %						
≤9	18.53	28.42	20.57	20.57	16.09	24.27
>9-16	30.40	28.04	27.78	30.46	27.87	29.38
>16-29	29.60	28.12	30.03	27.4	32.76	27.19
>29	21.47	15.43	21.62	18.21	23.28	19.16
Inpatient visits, mean (SD)	0.58 (0.78)	0.52 (0.62)	0.51 (0.67)	0.50 (0.64)	0.47 (0.67)	0.43 (0.56)
ED visits	0.60 (1.30)	0.42 (0.91)	0.70 (1.24)	0.53 (1.05)	0.67 (1.23)	0.48 (0.89)
No. of drugs, %						
≤6	18.27	16.41	18.02	15.07	16.38	12.59
>6-9	28.67	25.53	25.08	24.42	25.00	28.83
>9-13	25.07	28.95	25.53	29.41	31.61	32.30
>13	28.00	29.10	31.38	31.10	27.01	26.28

Abbreviations: ACE, angiotensin-converting enzyme; ARB, angiotensin II receptor blocker; CHA_2_DS_2_-VASc, congestive heart failure, hypertension, age ≥75 years (doubled), diabetes, stroke/transient ischemic attack/thromboembolism (doubled), vascular disease (prior myocardial infarction, peripheral artery disease, or aortic plaque), age 65-75 years, sex category (female); ED, emergency department; HAS-BLED, hypertension, abnormal renal and liver function, stroke, bleeding, labile international normalized ratio, elderly, and drugs or alcohol; NSAID, nonsteroidal anti-inflammatory drug; SSRI, selective serotonin reuptake inhibitor.

^a^ Includes 17 comorbidities, with higher numbers indicating higher comorbidity burden.

^b^ Risk score for stroke risk in nonvalvular atrial fibrillation that ranges between 0 and 9, with higher scores indicating higher stroke risk

^c^ Risk score for major bleeding while the patient is receiving anticoagulants that ranges from 0 to 9, with higher scores indicating higher major bleeding risk.

**Table 2.** Primary Analysis of Concomitant Direct Oral Anticoagulant Use With Verapamil or Diltiazem vs Amlodipine or Metoprolol Active Comparators

Bleeding categories	HR (95% CI)		
	Dabigatran	Rivaroxaban	Apixaban
**Verapamil or diltiazem vs amlodipine**			
Overall bleeding	1.52 (1.05-2.20)^a^	0.99 (0.71-1.38)	0.89 (0.49-1.63)
Overall GI bleeding	2.16 (1.30-3.60)^a^	0.64 (0.37-1.09)	0.70 (0.25-1.99)
Major/moderate bleeding	2.27 (0.97-5.29)	1.23 (0.65-2.35)	1.57 (0.35-7.16)
Major/moderate GI bleeding	2.27 (0.72-7.11)	0.51 (0.17-1.53)	2.17 (0.11-43.08)
Minor bleeding	1.56 (1.07-2.27)^a^	0.95 (0.68-1.35)	0.87 (0.47-1.63)
GI minor bleeding	2.16 (1.29-3.63)^a^	0.62 (0.35-1.09)	0.70 (0.25-1.99)
**Verapamil or diltiazem vs metoprolol**			
Overall bleeding	1.43 (1.02-2.00)^a^	0.76 (0.55-1.06)	0.78 (0.45-1.36)
Overall GI bleeding	2.32 (1.42-3.79)^a^	0.72 (0.42-1.22)	0.86 (0.40-1.86)
Major/moderate bleeding	3.32 (1.54-7.16)^a^	0.99 (0.50-1.98)	1.46 (0.33-6.41)
Major/moderate GI bleeding	5.49 (1.67-18.03)^a^	0.73 (0.23-2.25)	0.42 (0.02-8.71)
Minor bleeding	1.38 (0.98-1.95)	0.75 (0.54-1.06)	0.67 (0.37-1.21)
GI minor bleeding	2.33 (1.42-3.82)^a^	0.72 (0.42-1.24)	0.86 (0.40-1.86)

Abbreviations: GI, gastrointestinal; HR, hazard ratio.

^a^ Statistically significant at *P* < .0.5.

Similarly, patients receiving DOACs with verapamil or diltiazem vs metoprolol concomitantly differed, although less so, on characteristics (Table 3). After IPTW methods were applied (eTable 6 in the Supplement), all standardized differences were less than 10%, showing good balance between the groups. The number of events and crude event rates are reported in eTable 8 in the Supplement. Overall bleeding rates in the verapamil or diltiazem vs metoprolol groups were not statistically significantly different in the rivaroxaban (234.7 v 314.6 per 1000 person-years) and the apixaban (170.5 vs 219.4 per 1000 person-years) groups. In dabigatran users, overall bleeding rates were increased (291.3 vs 199.7 per 1000 person-years). Among patients receiving dabigatran, there were statistically significantly increased HRs of all bleeding categories except minor bleeding (Table 2): overall (HR, 1.43; 95% CI, 1.02-2.00), overall GI (HR, 2.32; 95% CI, 1.42-3.79), major/moderate (HR, 3.32; 95% CI, 1.54-7.16), major/moderate GI (HR, 5.49; 95% CI, 1.67-18.03), and minor GI (HR, 2.33; 95% CI, 1.42-3.82). All sensitivity analyses produced similar results of 50% to 100% increased hazard rates associated with dabigatran user but not apixaban or rivaroxaban use (eTable 9 and eTable 10 in the Supplement).

**Table 3.** Baseline Characteristic of Cohort of Direct Oral Anticoagulants With Verapamil or Diltiazem and With Metoprolol Use

Patient characteristics	Dabigatran		Rivaroxaban		Apixaban	
	Verapamil or diltiazem (n = 764)	Metoprolol (n = 1334)	Verapamil or diltiazem (n = 681)	Metoprolol (n = 1287)	Verapamil or diltiazem (n = 348)	Metoprolol (n = 603)
**Demographic**						
Age, %, y						
<65	56.94	56.37	62.70	62.63	56.61	58.71
65 to <70	12.70	10.94	12.33	13.75	15.52	13.93
70 to <75	11.13	12.67	9.69	10.41	12.64	13.43
75 to <80	10.60	10.64	10.13	8.94	9.48	9.12
80 to <85	7.07	7.20	3.82	3.57	4.02	3.81
≥85	1.57	2.17	1.32	0.70	1.72	1.00
Sex, %						
Women	46.47	39.58	44.79	38.46	47.99	42.45
Men	53.53	60.42	55.21	61.54	52.01	57.55
**Medical history, %**						
Diabetes without complication	25.92	26.39	23.64	26.03	29.31	24.71
Diabetes with complication	4.71	4.12	5.14	5.52	4.89	5.64
Hypertension	79.32	78.86	80.47	81.97	83.33	84.58
Hypercholesterolemia	54.19	61.32	58.74	66.67	64.08	69.49
Liver failure						
Mild	3.80	2.40	2.94	3.65	4.02	4.48
Moderate or severe	0.00	0.15	85.02	86.01	0.29	0.17
Cancer						
Solid	15.45	15.37	14.98	13.99	17.24	16.92
Fluid	1.31	1.35	1.17	1.32	1.44	0.50
Metastatic	1.44	1.35	1.03	0.39	0.57	1.49
Obesity	13.35	11.99	14.68	15.15	18.97	16.92
Peptic ulcer disease	0.79	0.37	0.73	0.31	0.00	0.33
Prior hospitalization for bleeding	9.03	8.17	8.81	9.32	12.36	10.45
Smoking	8.77	9.00	12.04	10.49	11.21	9.29
Cardiovascular disease						
Acute myocardial infarction						
Past 1-30 d	2.75	4.42	2.94	5.28	1.44	4.48
Past 31-180 d	1.44	2.40	1.76	3.19	3.45	2.49
Past 181-365 d	1.83	1.57	1.76	2.41	0.86	1.82
Coronary revascularization	8.90	9.07	5.73	8.62	10.06	8.79
Heart failure						
Hospitalized	9.95	11.62	8.22	9.40	7.47	8.29
Outpatient	10.86	14.77	14.10	13.68	10.34	12.60
Coronary artery disease	27.36	39.13	25.84	40.71	32.18	43.28
Other ischemic heart disease, d	25.13	36.43	22.76	37.92	29.89	40.63
Stroke						
Past 1-30 d	2.62	1.42	2.50	2.56	2.87	2.49
Past 31-180 d	1.31	1.50	1.62	1.79	3.16	1.49
Past 181-365 d	0.79	0.82	0.73	0.70	0.57	1.00
Other cerebrovascular disease	3.01	2.40	3.67	3.42	3.45	3.15
Transient ischemic attack	2.88	2.85	2.50	3.03	4.60	3.48
Cardioversion	15.84	18.82	13.66	17.72	19.83	18.57
Falls	1.31	1.42	2.20	1.94	2.59	2.49
Fractures	4.84	3.88	5.14	5.76	6.60	6.15
Syncope	6.15	8.10	7.34	7.38	7.76	9.12
Walker use	1.57	1.72	2.79	1.40	3.16	2.65
Charlson Comorbidity Index, mean (SD)^a^	1.52 (1.79)	1.48 (1.75)	1.50 (1.79)	1.44 (1.59)	1.62 (1.71)	1.65 (1.83)
CHA_2_DS_2_-VASc score^b^						
0-1	21.20	20.39	23.64	21.21	17.53	15.59
2	20.68	17.17	22.47	19.27	18.68	22.39
3	19.37	20.69	18.21	20.36	22.99	20.23
≥4	38.74	41.75	35.68	39.16	40.8	41.79
HAS-BLED score^c^						
0-1	35.73	37.41	38.77	34.97	28.16	29.68
2	39.27	36.43	36.27	38.62	41.95	39.80
3	19.50	20.39	19.82	21.29	22.70	23.71
≥4	5.50	5.77	5.14	5.13	7.18	6.80
**Medication use, %**						
General						
Estrogen therapy	6.68	4.50	4.55	4.35	4.60	6.97
Histamine_2_ antagonists	3.27	2.62	4.55	3.57	4.02	3.98
NSAID	21.86	21.51	23.35	25.17	27.59	23.22
Proton pump inhibitor	24.48	24.81	28.34	29.37	26.44	29.19
SSRI antidepressant	13.74	11.99	14.39	12.20	12.64	12.77
Cardiovascular						
ACE inhibitor	30.63	36.81	29.07	32.56	33.62	31.51
ARB	21.86	23.69	21.88	25.33	22.99	29.52
Antiarrhythmic	34.55	35.61	40.09	31.24	37.93	31.18
Other anticoagulant	2.09	1.20	0.73	0.47	0.57	0.50
Antiplatelet	9.82	14.54	11.60	15.31	8.91	19.40
β-blocker	25.52	14.92	21.88	12.74	23.28	12.27
Digoxin	16.10	12.37	11.75	9.01	16.09	10.45
Diuretics						
Loop	17.54	16.27	16.74	15.00	18.68	16.42
Potassium sparing	8.25	7.35	6.61	5.91	7.76	5.97
Thiazide and other	31.41	31.93	29.96	29.53	35.06	29.52
Nitrate	4.71	9.97	6.90	9.87	6.61	11.77
Statin	50.52	59.67	47.87	60.22	54.31	59.54
Fibrate	4.06	7.72	4.11	5.21	5.17	4.31
Other antihyperlipidemic	7.72	12.52	7.05	9.95	6.61	10.12
Diabetes related						
Insulin	3.01	3.97	3.67	5.05	5.75	4.48
Metformin	15.31	17.32	13.95	15.62	18.68	13.76
Sulfonylurea	7.20	6.82	6.75	7.54	8.62	5.14
Other	8.25	9.60	7.49	8.39	8.05	7.13
Metabolic inhibitor						
Amiodarone	7.07	10.72	9.10	8.47	9.20	8.46
Dronedarone	9.42	11.77	10.28	7.46	9.20	7.79
Azole antifungal	17.41	18.52	17.33	16.78	20.11	17.74
Metabolic inducer						
Carbamazepine	0.26	0.22	0.29	0.08	0.00	0.50
Phenytoin	0.39	0.00	0.15	0.16	0.00	0.00
Phenobarbital	0.13	0.00	0.15	0.00	0.00	0.00
**Prescriber specialty**						
Internal medicine	11.52	14.47	12.19	14.45	10.06	13.27
Family practice	10.34	9.97	8.08	9.32	12.64	10.28
Geriatric medicine	0.00	0.07	0.00	0.00	0.00	0.00
Cardiology	39.66	40.33	41.85	38.46	35.92	40.63
Critical care medicine	0.00	0.15	0.29	0.08	0.00	0.00
Hematology	0.13	0.00	0.15	0.08	0.00	0.17
Oncology	0.26	0.07	0.15	0.00	0.00	0.17
**Health care use**						
Outpatient visits, %						
≤9	18.32	21.89	19.53	22.07	14.94	20.73
>9-16	30.76	30.36	27.46	30.38	28.74	29.35
>16-29	29.58	32.01	30.54	29.99	33.91	29.68
>29	21.34	15.74	22.47	17.56	22.41	20.23
Inpatient visit, mean (SD)	0.60 (0.79)	0.60 (0.70)	0.53 (0.74)	0.55 (0.65)	0.46 (0.65)	0.48 (0.61)
ED visits	0.62 (1.33)	0.50 (0.95)	0.73 (1.26)	0.66 (1.19)	0.67 (1.22)	0.60 (1.02)
No. of prescriptions, %						
≤6	17.80	21.36	17.03	19.19	15.52	17.91
>6-9	27.49	26.69	24.38	28.90	23.85	27.03
>9-13	26.44	28.34	26.87	25.87	30.75	29.19
>13	28.27	23.61	31.72	26.03	29.89	25.87

Abbreviations: ACE, angiotensin-converting enzyme; ARB, angiotensin II receptor blocker; CHA_2_DS_2_-VASc, congestive heart failure, hypertension, age ≥75 years (doubled), diabetes, stroke/transient ischemic attack/thromboembolism (doubled), vascular disease (prior myocardial infarction, peripheral artery disease, or aortic plaque), age 65-75 years, sex category (female); ED, emergency department; HAS-BLED, hypertension, abnormal renal and liver function, stroke, bleeding, labile international normalized ratio, elderly, and drugs or alcohol; NSAID, nonsteroidal anti-inflammatory drug; SSRI, selective serotonin reuptake inhibitor.

^a^ Includes 17 comorbidities, with higher numbers indicating higher comorbidity burden.

^b^ Risk score for stroke risk in nonvalvular atrial fibrillation that ranges between 0 and 9, with higher scores indicating higher stroke risk.

^c^ Risk score for major bleeding while the patient is receiving anticoagulants that ranges from 0 to 9, with higher scores indicating higher major bleeding risk.

## Discussion

In our study of patients with no history of kidney disease receiving DOACs who had received a standard dose of each drug, use of dabigatran with concomitant verapamil or diltiazem was associated with an increased risk of bleeding compared with the combination of DOACs with amlodipine or metoprolol. There were no statistically significant findings for comparisons within groups using rivaroxaban or apixaban with verapamil or diltiazem vs the active comparison groups. To our knowledge, these findings provide the first real-world evidence of the pharmacokinetic drug-drug interactions between these medications and suggest that caution may be necessary when coprescribing dabigatran with common antihypertensive medications—verapamil and diltiazem—and possibly other moderate to strong P-gp inhibitors regardless of kidney function.

Dabigatran is a prodrug that undergoes efflux by P-gp and subsequent elimination via the intestinal tract or other elimination organs and is not a substrate for CYP450 enzymes. Thus, inhibition of P-gp increases the bioavailability of the prodrug, leading to increased serum concentrations of the active metabolite.^28^ Verapamil and diltiazem are moderate to strong P-gp inhibitors along with other common medications, such as amiodarone, proton-pump inhibitors, some antidepressants, and azole antifungals.^29^ In US Food and Drug Administration briefing documents, verapamil, used as a reference agent for drug-drug interactions, was reported to increase the area under the curve of dabigatran by over 143% with a single concomitant dose and by 18% when separated by a 2-hour dosing interval. In the phase 3 RE-LY clinical trial, P-gp inhibition led to an increase in major bleeding event rates (4.12% vs 2.96% for 150 mg dose; 3.99% vs 2.38% for 110 mg dose) though these findings were not statistically significant given the sample size in the trial.^30^ These figures represented a relative increase in bleeding risk of 39% to 68%, which are consistent with the findings from this study.

Current US Food and Drug Administration prescribing information for dabigatran does not recommend a dose reduction in the presence of P-gp inhibitors unless kidney function is compromised. A dose reduction to 75 mg twice daily is recommended for dabigatran when coadministered with a P-gp only in patients with creatinine clearance between 30 and 50 mL/min and dabigatran is not recommended to be coadministered with a P-gp inhibitor in patients with creatinine clearance less than 30 mL/min.^19^ Meanwhile, the European Medicines Agency recommendations and clinical guidance suggest a dabigatran dose reduction to 110 or 75 mg when given with verapamil regardless of kidney function.^31^ In contrast to international settings, the dabigatran, 110 mg, twice-daily dose is not approved for stroke prevention in patients in the US with nonvalvular atrial fibrillation. Comparatively, apixaban requires no dose adjustment based on kidney function alone (but can be adjusted when age and body weight are considered) and the rivaroxaban dose should be reduced to 15 mg once daily if creatinine clearance is less than 50 mg/mL.^20,21^ Apixaban and rivaroxaban labels include prescribing recommendation changes only if combined P-gp and strong CYP3A4 inhibitors or inducers are to be used concomitantly. However, rivaroxaban had higher bleeding event rates compared with dabigatran in our study, which is consistent with a network meta-analysis comparing DOACs.^32^ In that meta-analysis, apixaban had consistently lower bleeding event rates among all DOACs. Thus, treatment recommendations and changes should consider both the effect of the interaction and the baseline risk of adverse events.

### Strengths and Limitations

To our knowledge, this is the first real-world observational study that evaluated the drug-drug interaction between DOACs and verapamil or diltiazem vs active comparators. This design may help to reduce indication and healthy user biases, provide some control for disease severity, and also provide useful information for patients and prescribers when they need to consider an alternative treatment.

However, there are still several limitations in our study. First, we only looked at the use of verapamil, diltiazem, amlodipine, and metoprolol before DOAC initiation, and required 90 days of cumulative use of these medications during the previous 6-month period with strict criteria for censoring at discontinuation or switching of any study medication (DOACs or antihypertensives). While this approach substantially reduced our sample size, it helped to create a more homogeneous population of patients receiving long-term therapy with each medication and provided strong exposure definitions. Second, owing to the small sample size, we combined data on verapamil and diltiazem. Although this combination is not ideal from a study design perspective, both verapamil and diltiazem are categorized as combined P-gp inhibitors and moderate CYP3A4 inhibitors, have similar on-label and off-label indications, and are expected to behave similarly in terms of drug-drug interaction mechanisms.

As in all other observational studies, the potential of unmeasured confounding may not be avoided; however, we minimized this by having an active comparator study design in addition to IPTW methods and a large number of covariates. Owing to the limitations of claims data, we could not observe when patients administered the drugs; thus, our conclusions were based on the assumption that patients followed what had been recommended by physicians. Our exposure definition required continuous adherence with a small gap window (7-day) to ensure exposure.^33,34^ Likewise, the claims database would not include information on when patients acquired prescriptions not paid for by their insurance coverage, such as over-the-counter or sample medications,^35,36,37^ but this information is not expected to be imbalanced between treatment groups and would likely not affect the study results. Our results were consistent in direction and magnitude to several sensitivity analyses including use of standardized mortality ratio weighting in place of IPTW, use of a 15-day gap censor criterion, outcome measurement using all primary and secondary diagnoses, and separating verapamil and diltiazem.

## Conclusions

The drug-drug interaction between DOACs and the P-pg inhibitors verapamil and diltiazem appears to show evidence of an increased rate of bleeding among patients receiving dabigatran but not apixaban or rivaroxaban compared with active comparator control groups in patients with normal kidney function. To our knowledge, this is the first real-world study to show this outcome and the findings contrast with current US prescribing recommendations. Clinicians and patients may need to consider alternative DOAC therapy other than dabigatran during concomitant use of moderate to strong P-gp inhibitors regardless of kidney function or find medications that do not interact if dabigatran must be used.

## Notes

Supplement. **eTable 1.** ICD 9-CM Codes for Comorbidities **eTable 2.** ICD 9 Codes for HAS-BLED Score **eTable 3.** ICD 9 Codes for CHA2DS2-VASc Score **eTable 4.** ICD-9-CM Codes for Study Outcomes **eReferences.** **eFigure 1.** Cohort Selection and Attrition **eTable 5.** Standardized Percent Difference of Cohort’s Baseline Characteristic After Inverse Probability of Treatment Weighting When Comparing Verapamil/Diltiazem Versus Amlodipine or Metoprolol Among Each DOAC Cohort **eTable 6.** Standardized Difference of Cohort’s Baseline Characteristic After Inverse Probability of Treatment Weighting When Comparing DOAC Together Among Cohort of Verapamil/Diltiazem, Amlodipine or Metoprolol **eTable 7.** Number and Incident Rate of Adverse Bleeding Events Among Concomitant Users Of DOACs With Verapamil/Diltiazem and With Amlodipine **eTable 8.** Number and Incident Rate of Adverse Bleeding Events Among Concomitant Users Of DOACs With Verapamil/Diltiazem and With Metoprolol **eTable 9.** Hazard Ratio of Adverse Bleed Events Among Concomitant Users of Direct Oral Anticoagulant and Verapamil/Diltiazem Versus Amlodipine **eTable 10.** Hazard Ratio of Adverse Bleed Events Among Concomitant Users of Direct Oral Anticoagulant and Verapamil/Diltiazem Versus Metoprolol
