# Supplementary material for: Association of Oral Anticoagulants and Verapamil or Diltiazem With Adverse Bleeding Events in Patients With Nonvalvular Atrial Fibrillation and Normal Kidney Function
Source: JAMA Netw Open. 2020 Apr 24;3(4):e203593. doi: 10.1001/jamanetworkopen.2020.3593 (PMC7182798; doi:10.1001/jamanetworkopen.2020.3593)
Supplement: Supplement. — eTable 1. ICD 9-CM Codes for Comorbidities eTable 2. ICD 9 Codes for HAS-BLED Score eTable 3. ICD 9 Codes for CHA2DS2-VASc Score eTable 4. ICD-9-CM Codes for Study Outcomes eReferences. eFigure 1. Cohort Selection and Attrition eTable 5. Standardized Percent Difference of Cohort’s Baseline Characteristic After Inverse Probability of Treatment Weighting When Comparing Verapamil/Diltiazem Versus Amlodipine or Metoprolol Among Each DOAC Cohort eTable 6. Standardized Difference of Cohort’s Baseline Characteristic After Inverse Probability of Treatment Weighting When Comparing DOAC Together Among Cohort of Verapamil/Diltiazem, Amlodipine or Metoprolol eTable 7. Number and Incident Rate of Adverse Bleeding Events Among Concomitant Users Of DOACs With Verapamil/Diltiazem and With Amlodipine eTable 8. Number and Incident Rate of Adverse Bleeding Events Among Concomitant Users Of DOACs With Verapamil/Diltiazem and With Metoprolol eTable 9. Hazard Ratio of Adverse Bleed Events Among Concomitant Users of Direct Oral Anticoagulant and Verapamil/Diltiazem Versus Amlodipine eTable 10. Hazard Ratio of Adverse Bleed Events Among Concomitant Users of Direct Oral Anticoagulant and Verapamil/Diltiazem Versus Metoprolol [file jamanetwopen-3-e203593-s001.pdf]

Supplementary Online Content

Pham P, Schmidt S, Lesko L, Lip GYH, Brown JD. Association of oral anticoagulants and verapamil or diltiazem with adverse bleeding events in patients with nonvalvular atrial fibrillation and normal kidney function. *JAMA Netw Open*. 2020;3(4):e203593. doi:10.1001/jamanetworkopen.2020.3593

eTable 1. ICD 9-CM Codes for Comorbidities

eTable 2. ICD 9 Codes for HAS-BLED Score

eTable 3. ICD 9 Codes for CHA2DS2-VASc Score

eTable 4. ICD-9-CM Codes for Study Outcomes

eReferences

eFigure 1. Cohort Selection and Attrition

eTable 5. Standardized Percent Difference of Cohort's Baseline Characteristic After Inverse Probability of Treatment Weighting When Comparing Verapamil/Diltiazem Versus Amlodipine or Metoprolol Among Each DOAC Cohort

eTable 6. Standardized Difference of Cohort's Baseline Characteristic After Inverse Probability of Treatment Weighting When Comparing DOAC Together Among Cohort of Verapamil/Diltiazem, Amlodipine or Metoprolol

eTable 7. Number and Incident Rate of Adverse Bleeding Events Among Concomitant Users Of DOACs With Verapamil/Diltiazem and With Amlodipine

eTable 8. Number and Incident Rate of Adverse Bleeding Events Among Concomitant Users Of DOACs With Verapamil/Diltiazem and With Metoprolol

eTable 9. Hazard Ratio of Adverse Bleed Events Among Concomitant Users of Direct Oral Anticoagulant and Verapamil/Diltiazem Versus Amlodipine

eTable 10. Hazard Ratio of Adverse Bleed Events Among Concomitant Users of Direct Oral Anticoagulant and Verapamil/Diltiazem Versus Metoprolol

This supplementary material has been provided by the authors to give readers additional information about their work.

© 2020 Pham P et al. *JAMA Network Open.*

eTable 1: ICD 9-CM codes for comorbidities

Comorbidity	ICD-9 codes	Ref
**For stratification**		
Cancer<br>*-Solid tumor cancer*<br>*-Blood cancer*<br>*-Metastatic cancer*	 140-195<br>200-208<br>196-199	[21]
Renal disease<br>*-Acute*<br>*-Chronic*<br>*Stage 1*<br>*Stage 2_mild*<br>*Stage 3_moderate*<br>*Stage 4_severe*<br>*Stage 5*	 584.5-584.9<br><br>585.1<br>585.2<br>585.3<br>585.4<br>585.5	 [8]<br>[9]
ESRD<br>*-Dialysis*	585.6<br>V45.1, V56.0, V56.1<br>39.95,54.93,54.98,38.95,39.27,<br>90935, 90937,90945,90947, 90951-90970,<br>90989, 90993, 90997, 90999, 90918-90925,<br>93990	[8,10,12,13]
Liver disease<br>*-Mild*<br><br><br><br>*Mediate or severe*	 070.22, 070.23, 070.32, 070.33,<br>070.44, 070.54, 070.6, 070.9,<br>570.x, 571.x, 573.3, 573.4,<br>573.8, 573.9, V42.7<br>456.0–456.21, 572.2–572.8	[14]
Diabetes<br>-without chronic complication<br>-with chronic complication	 250.0–250.3, 250.8, 250.9<br>250.4–250.7	[14]
Hypertension	401.x-405.x	[14]
Hyperlipidemia	272.0, 272.1, 272.2, 272.3, 272.4	[15]
Obesity	278.00	[14]
Gastric ulcer	531, 532, 533, 534	[21]
**For exclusion criteria**		[16]
Mitral stenosis or prosthetic heart valve	394.0, 394.2, 396.0, 396.1, 396.8, V43.3, or V42.2	
Mitral or aortic valve repair or replacement	35.10 to 35.14 or 35.20 to 35.28	[16]

© 2020 Pham P et al. *JAMA Network Open.*

Joint replacement	0080 0081 0082 0083 0084 8141 8142 8143 8144 8146 8147 8154 8155 0070 0071 0072 0073 0074 0075 0076 0077 0085 0086 0087 8151 8152 8153 8169 8148 8149 8156 8157 8159 8171 8172 8173 8174 8175 8179 8180 8181 8182 8183 8184 8185 8186 8187 8188 8197	[21]
**For confounding adjusting**		
Cardiovascular disease		
-Deep vein thrombosis	451.x, 453.x	[17]
-Pulmonary embolism	415.x	[17]
-Congestive Heart Failure	398.91, 402.01, 402.11, 402.91, 404.01, 404.03, 404.11,<br>404.13, 404.91, 404.93,425.4–425.9, 428.x	[14]
-Coronary Artery Disease	410.00, 410.01, 410.02, 410.10, 410.11, 410.12, 410.20, 410.21, 410.22, 410.30, 410.31, 410.32, 410.40, 410.41, 410.42, 410.50, 410.51, 410.52, 410.60, 410.61, 410.62, 410.70, 410.71, 410.72, 410.80, 410.81, 410.82, 410.90, 410.91, 410.92, 411.0, 411.1, 411.81, 411.89, 412, 413.0, 413.1, 413.9, 414.00, 414.01, 414.02, 414.03, 414.04, 414.05, 414.06, 414.07, 414.8, 414.9, V45.81, V45.82	[10]
-Acute MI	410	[1]
-Coronary revascularization	36.01-36.07, 36.09, 00.66, 36.1x	[11]
-Heart failure	402.01, 402.11, 402.91, 404.01, 404.03, 404.11, 404.13, 404.91, 404.93, 428.0, 428.1, 428.20, 428.21, 428.22, 428.23, 428.30, 428.31, 428.32, 428.33, 428.40, 428.41, 428.42, 428.43, 428.9	[10]
-Other ischemic heart disease	410.00, 410.01, 410.02, 410.10, 410.11, 410.12, 410.20, 410.21, 410.22, 410.30, 410.31, 410.32, 410.40, 410.41, 410.42, 410.50, 410.51, 410.52, 410.60, 410.61, 410.62, 410.70, 410.71, 410.72, 410.80, 410.81, 410.82, 410.90, 410.91, 410.92, 411.0, 411.1, 411.81, 411.89, 412, 413.0, 413.1, 413.9, 414.00, 414.01, 414.02, 414.03, 414.04, 414.05, 414.06, 414.07, 414.12, 414.2, 414.3, 414.8, 414.9	[15]
-Stroke	433.x1, 434.x1, 436, 430, 431	[7]
-Other cerebrovascular disease	430.x - 438.x	[21]
-TIA	435.x	[7]

© 2020 Pham P et al. *JAMA Network Open.*

-Cardioablation	93650, 93651, 93652, 93653, 93654, 93656, 93655, 93657, 93462	[18]
-Cardioversion	99.61,99.62, 99.69<br>92960, 92961	[19]
Other medical conditions		
-Falls	E880-E888, E968.1, E987.0, E987.1, E987.2, E987.9, V15.88	[21]
-Fracture	820–821, 813, 812, 805	[21]
-Syncope	780.2	[20]
-Walker use	E0100, E0105, E0110, E0111, E0112, E0113, E0114, E0116, E0117, E0118, E0130, E0135, E0140, E0141, E0143, E0144, E0147, E0148, E0149, E0153, E0154, E0155, E0156, E0157, E0158	[22]

© 2020 Pham P et al. *JAMA Network Open.*

eTable 2: ICD 9 Codes for HAS-BLED score [7]

**HAS-BLED**	**Score**	**ICD-9-CM codes**
Hypertension	1	401.x-405.x
Abnormal renal function	1	585.3, 585.4
Abnormal liver function	1	070.x, 570, 571.x, 572.x, 573.0-5,
		V42.7
Stroke	1	430, 431, 433.x1, 434.x1, 436
Bleeding history or	1	280.x-287.x, 430, 431, 432.x, 852.x,
predisposition		853.x, 456.0, 456.20, 530.21, 530.7,
		530.82, 531.0x, 531.2x, 531.4x,
		531.6x, 532.0x, 532.2x, 532.4x,
		532.6x, 533.0x, 533.2x, 533.4x,
		533.6x, 534.0x, 534.2x, 534.4x,
		534.6x, 535.01, 535.11, 535.21,
		535.31, 535.41, 535.51, 535.61,
		535.71, 537.83, 537.84, 562.02,
		562.03, 562.12, 562.13, 568.81, 569.3,
		569.85, 578.x, 423.0, 459.0, 596.7,
		599.71, 719.1x, 784.8, 786.3
Elderly (>65)	1	
Antiplatelet or NSAID use	1	
Alcoholism	1	291.x, 303.x, 305.0x, 357.5, 425.5,
		535.3x, 571.0-571.3, 760.71, 980.0,
		V11.3
Maximum score	8	

© 2020 Pham P et al. *JAMA Network Open.*

eTable 3: ICD 9 Codes for CHA2DS2-VASc score [7]

CHA_2DS_2-VASc	Score	ICD-9-CM codes
Congestive heart failure	1	428.x, 402.x1, 404.x1, 404.x3, 398.91
Hypertension	1	401.x-405.x
Diabetes mellitus	1	250.x, 357.2, 362.0x, 366.41
Stroke/TIA/TE	2	430, 431, 433.x1, 434.x1, 436, 435.x,
		444.x
Vascular disease	1	410-414, 443.8,443.9, 440.x
Age≥75	2	
Age 65-74	1	
Female sex	1	
Maximum score	9	

© 2020 Pham P et al. *JAMA Network Open.*

eTable 4: ICD-9-CM codes for study outcomes

Outcomes	ICD-9 codes	Reference
**Major bleeding**	*->Bleeding events in inpatient only and meet the definition for major bleeding: a bleeding event with (i) a critical site code, (ii) a transfusion, or (iii) death*	
Overall	Critical site: Intracranial: 430, 431, 432, 852.0, 852.2, 852.4, 853.0; Extracranial: 336.1, 363.6, 372.72, 376.32, 377.42, 379.23, 719.1, 729.92, 729.97, 423.0, 593.81, 772.5, 866.01, 866.02, 866.11, 866.12<br>Transfusions: a) ICD-9 PRC: 9903, 9904, 9905, 9906, 9907, 9909, b) HCPC: P9010, P9011, P9016, P9017, P9019, P9020	[1]
ICH	430, 431, 432, 852.0, 852.2, 852.4, 853.0	[1]
GI bleeding	*->Bleeding at GI site and need a transfusion or lead to death.*<br>455.2, 455.5, 455.8, 456.0x, 456.20, 530.7x, 530.82, 531.00, 531.01, 531.20, 531.21, 531.40, 531.41, 531.60, 531.61, 532.00, 532.01, 532.20, 532.21, 532.40, 532.41, 532.60, 532.61, 533.00, 533.01, 533.20, 533.21, 533.40, 533.41, 533.60, 533.61, 534.00, 534.01, 534.20, 534.21, 534.40, 534.41, 534.60, 534.61, 535.01, 535.11, 535.21, 535.31, 535.41, 535.51, 535.61, 537.83, 537.84, 562.02, 562.03, 562.12, 562.13, 568.81, 569.3× or 569.85, 569.86, 578.0x, 578.1x, 578.9×<br>(*combine these with codes for transfusion or death*)	[1]
**Moderate bleeding**	*->Bleeding events in inpatient or emergency department that do not meet the criteria for major bleeding*	
Overall	531.0x, 531.2x, 531.4x, 531.6x, 532.0x, 532.2x, 532.4x, 532.6x, 533.0x, 533.2x, 533.4x, 533.6x, 534.0x, 534.2x, 534.4x, 534.6x, 535.01, 535.11, 535.21, 535.31, 535.41, 535.51, 535.61, 537.83, 456.0, 456.20, 530.7,530.82, 578.0, 455.2, 455.5, 455.8, 562.02, 562.03, 562.12, 562.13, 568.81, 569.3, 569.85, 578.1, 578.9, 593.81, 599.7, 623.8, 626.2, 626.6, 423.0, 459.0, 568.81, 719.1x, 784.7, 784.8, 786.3	[1]
GI bleeding	455.2, 455.5, 455.8, 456.0x, 456.20, 530.7x, 530.82, 531.00, 531.01, 531.20, 531.21, 531.40, 531.41, 531.60, 531.61, 532.00, 532.01, 532.20, 532.21, 532.40, 532.41, 532.60, 532.61, 533.00, 533.01, 533.20, 533.21, 533.40, 533.41, 533.60, 533.61, 534.00, 534.01, 534.20, 534.21, 534.40, 534.41, 534.60, 534.61, 535.01, 535.11, 535.21, 535.31, 535.41, 535.51, 535.61, 537.83, 537.84, 562.02, 562.03, 562.12, 562.13, 568.81, 569.3× or 569.85, 569.86, 578.0x, 578.1x, 578.9×	
**Minor bleeding**	Bleeding events in outpatient office visits only.<br>Use same codes for moderate bleeding but in outpatients.	

© 2020 Pham P et al. *JAMA Network Open.*

**Acute MI**	410	[2-6]
**Stroke or systemic embolism**		[7]
Ischemic stroke	433.x1, 434.x1, or 436	
Hemorrhagic stroke	430, 431	
Systemic embolism	444.x	
TIA	435.x	

© 2020 Pham P et al. *JAMA Network Open.*

eREFERENCES

1. Graham DJ, Reichman ME, Wernecke M, et al. Stroke, Bleeding, and Mortality Risks in Elderly Medicare Beneficiaries Treated With Dabigatran or Rivaroxaban for Nonvalvular Atrial Fibrillation. *JAMA Intern Med.* Vol 176. United States2016:1662-1671.

2. Petersen LA, Wright S, Normand SL, Daley J. Positive predictive value of the diagnosis of acute myocardial infarction in an administrative database. *J Gen Intern Med.* Sep 1999;14(9):555-558.

3. Levy AR, Tamblyn RM, Fitchett D, McLeod PJ, Hanley JA. Coding accuracy of hospital discharge data for elderly survivors of myocardial infarction. *Can J Cardiol.* Nov 1999;15(11):1277-1282.

4. Austin PC, Daly PA, Tu JV. A multicenter study of the coding accuracy of hospital discharge administrative data for patients admitted to cardiac care units in Ontario. *Am Heart J.* Vol 144. United States2002:290-296.

5. Kiyota Y, Schneeweiss S, Glynn RJ, Cannuscio CC, Avorn J, Solomon DH. Accuracy of Medicare claims-based diagnosis of acute myocardial infarction: estimating positive predictive value on the basis of review of hospital records. *Am Heart J.* Vol 148. United States2004:99-104.

6. Choma NN, Griffin MR, Huang RL, et al. An algorithm to identify incident myocardial infarction using Medicaid data. *Pharmacoepidemiol Drug Saf.* Nov 2009;18(11):1064-1071.

7. Noseworthy PA, Yao X, Abraham NS, Sangaralingham LR, McBane RD, Shah ND. Direct Comparison of Dabigatran, Rivaroxaban, and Apixaban for Effectiveness and Safety in Nonvalvular Atrial Fibrillation. *Chest.* Vol 150. United States: A 2016 American College of Chest Physicians. Published by Elsevier Inc; 2016:1302-1312.

8. Waikar SS, Wald R, Chertow GM, et al. Validity of International Classification of Diseases, Ninth Revision, Clinical Modification Codes for Acute Renal Failure. *J Am Soc Nephrol.* Jun 2006;17(6):1688-1694.

9. OPTUM. Documentation & Coding of Chronic Kidney Disease (CKD). 2013. Available at https://www.cmocares.org/documents/diagnostic-guidelines/chronic-kidney/Chronic_Kidney_Disease_Reporting1.pdf. Assessed on Oct 01, 2017

10. 2011 Physician quality reporting system. Measure specifications manual for claims and registey reporting of individual measures. Available: https://www.cms.gov/Medicare/Quality-Initiatives-Patient-Assessment-Instruments/PQRS/downloads/2011_PhysQualRptg_MeasureSpecificationsManual_033111.pdf. Assessed on 01/10/2017

11. Epstein AJ, Polsky D, Yang F, Yang L, Groeneveld PW. Coronary Revascularization Trends in the United States, 2001-2008. JAMA. 2011;305(17):1769–1776. doi:10.1001/jama.2011.55

12. UnitedHealthcare. Dialysis services. Oxford Health Plans. Available at https://www.oxhp.com/secure/policy/dialysis_services.pdf. Assessed on Oct 01, 2017

13. Taneja C, Berger A, Inglese GW, et al. Can dialysis patients be accurately identified using healthcare claims data? *Perit Dial Int.* Sep-Oct 2014;34(6):643-651.

14. Quan H, Sundararajan V, Halfon P, et al. Coding algorithms for defining comorbidities in ICD-9-CM and ICD-10 administrative data. *Med Care.* Vol 43. United States2005:1130-1139.

15. Buccaneer AGDC. Chronic Condition Data Warehouse. 2012.Available at https://www.ccwdata.org/web/guest/condition-categories. Assessed on Oct 01, 2019

16. Brown JD, Shewale AR, Talbert JC. Adherence to Rivaroxaban, Dabigatran, and Apixaban for Stroke Prevention in Incident, Treatment-Naïve Nonvalvular Atrial Fibrillation. *Journal of managed care & specialty pharmacy.* 2016;22(11):1319-1329.

17. Tamariz L, Harkins T, Nair V. A systematic review of validated methods for identifying venous

© 2020 Pham P et al. *JAMA Network Open.*

thromboembolism using administrative and claims data. *Pharmacoepidemiol Drug Saf.* Jan 2012;21 Suppl 1:154-162.

18. Medtronic. Cathter ablation reimbursement guide. 2015.Available at http://www.medicalbillingcodings.org/2013/05/cardiology-cpt-codes-new-changes.html.Assessed on Oct 01, 2017

19. Noseworthy PA, Kapa S, Deshmukh AJ, et al. Risk of stroke after catheter ablation versus cardioversion for atrial fibrillation: A propensity-matched study of 24,244 patients. *Heart Rhythm.* Vol 12. United States: 2015 Heart Rhythm Society. Published by Elsevier Inc; 2015:1154-1161.

20. Schuur JD, Justice A. Measuring quality of care in syncope: case definition affects reported electrocardiogram use but does not bias reporting. *Acad Emerg Med.* Jan 2009;16(1):40-49.

21. Clinical Classifications Solfware (CCS) for ICD9-CM. Available: https://www.hcup-us.ahrq.gov/toolssoftware/ccs/AppendixASingleDX.txt. Assessed on 01/10/2017

22. HCPCS codes. Available at https://hcpcs.codes/e-codes/. Assessed on 01/10/2017

© 2020 Pham P et al. *JAMA Network Open.*

eFigure 1: Cohort selection and attrition

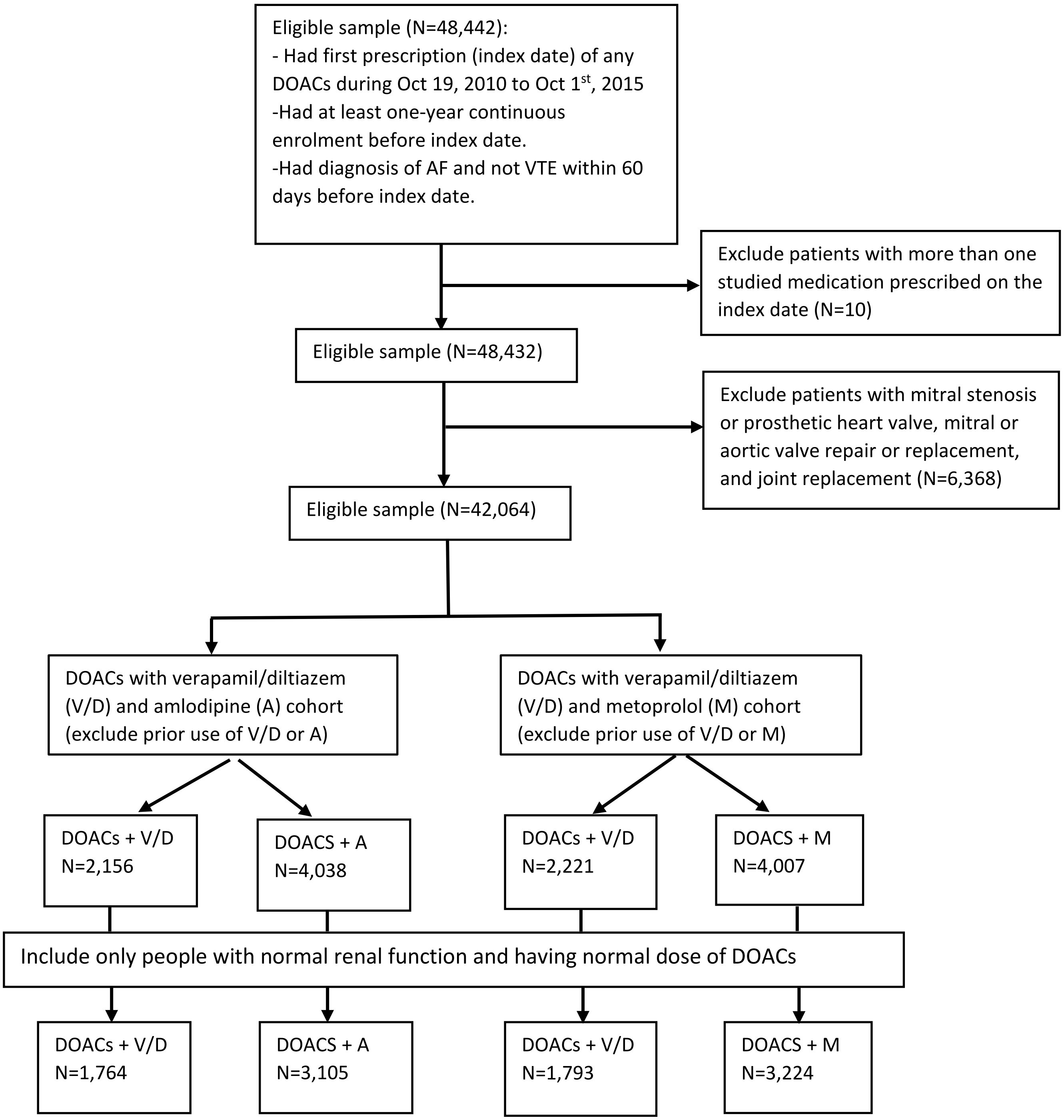

© 2020 Pham P et al. *JAMA Network Open.*

eTable 5: Standardized percent difference of cohort's baseline characteristic after inverse probability of treatment weighting when comparing verapamil/diltiazem versus amlodipine or metoprolol among each DOAC cohort. Differences <10% are considered to indicate well balance covariate distribution between comparison groups.						
	**Dabigatran**	**Rivaroxaban**	**Apixaban**	**Dabigatran**	**Rivaroxaban**	**Apixaban**
**Patients' characteristic**	V/D vs A	V/D vs A	V/D vs A	V/D vs M	V/D vs M	V/D vs M
**Demographic**						
Age, %						
<65	-3.05	1.62	-11.21	-1.52	0.10	-1.3
65-<70	-1.87	-3.03	1.02	0.78	-0.46	0.48
70-<75	2.10	-0.10	10.15	1.85	-0.07	0.82
75-<80	3.67	1.11	-1.04	-1.38	0.57	0.84
80-<85	1.97	-1.02	7.22	1.85	-0.50	-0.17
≥ 85	-1.57	1.24	5.31	-0.97	0.63	-0.09
Sex, %						
Female	-2.21	-2.16	-0.77	-1.35	1.17	3.57
**Medical history, %**						
General						
Diabetes w/o complication	-1.74	1.86	-5.59	4.37	0.61	3.77
Diabetes w/complication	-4.09	3.96	1.51	3.02	0.49	-0.10
Hypertension	3.34	2.36	3.79	-1.03	-0.37	-2.71
Hypercholesterolemia	3.99	1.81	-1.92	0.06	-1.10	1.55
Liver failure						
Mild	-0.90	2.25	1.49	5.42	-2.03	2.42
Moderate or severe	0.00	3.27	-4.77	4.37	0.00	-0.05
Cancer						
Solid	-5.05	-1.03	3.67	0.78	1.02	2.21
Fluid	1.31	1.01	-5.41	1.06	-2.60	-2.92
Metastatic	-1.85	2.18	1.37	-1.35	-0.80	2.52
Obesity	3.32	-4.65	-4.73	3.63	-0.27	2.81
Peptic ulcer disease	0.35	1.88	8.36	0.23	-0.58	6.50
Prior hospitalized bleeding event	-0.32	1.31	0.87	0.17	0.77	1.89
Smoking	-1.61	2.04	-2.01	-0.24	-0.78	-0.50
Cardiovascular disease						
Acute myocardial infarction						
Past 1-30 days	-1.41	-2.71	5.56	-3.37	3.01	9.76
Past 31-180 days	-1.11	-1.06	4.82	-1.25	1.85	3.04
Past 181-365 days	2.18	-3.97	4.58	-0.52	3.17	0.83
Coronary revascularization	0.59	0.07	1.26	0.58	0.25	1.33

© 2020 Pham P et al. *JAMA Network Open.*

Heart failure						
Hospitalized	-0.23	-0.35	-0.24	-0.68	1.77	0.12
Outpatient	-4.14	3.21	5.24	-6.89	1.74	2.70
Coronary artery disease	3.78	1.16	1.84	3.91	6.68	5.72
Other ischemic heart disease	3.83	0.23	3.14	4.66	6.67	5.13
Stroke						
Past 1-30 days	-0.36	3.04	1.46	-0.91	1.02	-2.66
Past 31-180 days	-6.22	-3.12	-1.43	-1.75	-7.02	-0.48
Past 181-365 days	-0.62	2.60	3.11	-1.84	0.38	4.43
Other cerebrovascular disease	-5.46	5.05	0.85	-2.01	1.23	-1.61
Transient ischemic attack (TIA)	-3.99	-2.31	0.69	2.64	1.28	-0.90
Cardioversion	3.17	-8.77	-2.76	-5.88	-3.28	1.86
Other medical conditions						
Falls	-5.05	2.84	8.58	2.95	-5.82	1.52
Fractures	-1.39	4.29	1.08	-1.94	0.99	-7.66
Syncope	-3.26	-2.38	-0.04	0.34	-0.54	-0.36
Walker use	-0.93	4.17	-0.84	-0.34	-0.24	2.08
CCI, Mean (SD)	-5.35	3.31	3.91	0.16	0.59	4.69
CHA_2DS_2-VASc Score						
0-1	-4.10	-3.55	-5.13	0.14	0.12	-2.06
2	4.26	6.03	0.11	5.78	-0.55	0.66
3	1.20	-5.90	4.66	-6.76	0.21	3.13
≥4	-2.96	5.29	0.78	-0.23	0.57	-2.60
HAS-BLED score						
0-1	-1.84	0.20	-5.22	-1.09	-1.12	1.47
2	0.77	0.51	0.52	2.90	-1.08	-5.94
3	-1.66	3.65	-8.36	-4.48	3.40	2.30
≥4	2.07	-3.52	9.94	2.34	-0.88	2.07
**Medication use, %**						
General						
Estrogen therapy	1.72	-1.52	3.09	1.98	-1.49	1.98
Histamine 2 antagonists	3.80	1.10	0.52	1.08	-1.38	5.81
NSAID	2.57	-1.72	1.02	-6.21	-0.81	4.21
Proton pump inhibitors	0.48	-0.15	0.47	-3.95	-1.31	5.19
SSRI antidepressants	1.09	5.67	-1.89	-7.56	-1.57	1.49
Cardiovascular						
ACE inhibitors	-2.66	-0.80	-2.80	0.83	1.87	-0.07
ARBs	2.61	1.37	4.68	-4.51	0.26	3.46
Antiarrhymics	-2.62	0.92	-2.60	-3.26	-1.24	-5.85
Other anticoagulants	-0.89	0.45	-0.72	-0.37	-0.10	0.10
Antiplatelet	2.32	-0.97	6.53	3.51	3.40	0.96
B-blocker	1.55	-0.70	6.56	2.47	0.03	1.67

© 2020 Pham P et al. *JAMA Network Open.*

Digoxin	1.46	3.55	-0.67	2.19	-0.19	0.20
Diuretics						
Loop	-3.29	3.16	-1.48	-6.00	1.51	-1.55
Potassium sparing	0.29	-4.18	1.68	-0.41	-1.65	4.11
Thiazide and other	0.79	0.81	1.51	-5.00	-3.39	0.47
Nitrates	-0.02	6.29	8.05	2.76	4.06	9.88
Statins	1.75	1.44	-2.13	-3.87	3.44	3.36
Fibrates	3.01	2.33	-0.02	-4.44	1.69	2.68
Other antihyperlipidemic	0.31	0.07	6.32	-6.00	1.51	-1.55
Diabetes related						
Insulin	-1.35	-2.28	-0.96	-0.07	-0.22	-0.47
Metformin	2.11	0.11	0.37	-3.54	0.22	2.36
Sulfonylurea	-0.82	-5.87	-1.78	1.04	-0.69	1.61
Other	-1.03	6.53	-6.23	3.14	1.10	2.15
Metabolic inhibitors						
Amiodarone	-2.80	0.59	4.67	-1.03	0.98	-1.18
Dronedarone	-0.50	-0.31	-0.63	-4.94	0.38	-6.19
Azole antifungals	0.95	1.70	0.59	1.00	-0.39	-1.21
Metabolic inducers						
Carbamazepine	-0.83	1.93	6.82	0.44	-1.00	7.96
Phenytoin	-5.33	2.96	-4.77	-5.31	-1.27	0.00
Phenobarbital	0.13	0.17	0.00	-3.06	-3.21	0.00
Prescriber specialty				0.44	-1.00	7.96
Internal medicine	0.84	-10.95	1.87	1.51	-0.38	-2.92
Family practice	-0.43	-1.39	2.25	-0.51	-1.04	0.71
Geriatric medicine	3.15	3.27	0.00	3.09	0.00	0.00
Cardiology	2.00	6.04	-1.89	-3.70	1.58	1.66
Critical care medicine	3.15	-4.49	4.82	4.37	-0.45	0.00
Hematology	-1.65	-3.18	0.00	-3.06	-0.04	4.59
Oncology	-4.35	0.19	0.00	-0.20	-3.21	4.59
**Healthcare utilization**						
Outpatient visits, %						
≤ 9	2.26	4.23	-0.46	-2.87	2.44	-6.03
>9-16	0.42	-7.74	0.68	0.25	0.40	1.72
>16-29	-2.64	0.25	-1.45	2.11	-2.51	0.35
>29	0.11	4.46	1.34	0.21	-0.02	3.66
Inpatient visits, Mean (SD)	-1.67	-1.28	-0.82	1.70	-2.39	2.49
ER visits	-2.39	2.41	2.99	5.35	-2.32	-1.32
Number of Rx, %						
≤ 6	-0.14	2.70	-2.78	2.05	4.08	-5.93
>6-9	-1.37	-2.45	-1.11	-5.42	-0.34	3.16
>9-13	4.01	-1.11	-0.70	-0.71	-1.68	0.74
>13	-2.45	1.25	4.02	4.50	-1.44	1.37
V/D: verapamil/diltiazem; A: amlodipine;						

© 2020 Pham P et al. *JAMA Network Open.*

CCI: Charlson comorbidity index; NSAID: nonsteroidal anti-inflammatory drugs; SSRI: selective serotonin reuptake inhibitors; ACE inhibitors: Angiotensin-converting enzyme inhibitors; ARB: Angiotensin II receptor blockers; ER: emergency room

© 2020 Pham P et al. *JAMA Network Open.*

eTable 6: Standardized difference of cohort's baseline characteristic after inverse probability of treatment weighting when comparing DOAC together among cohort of verapamil/diltiazem, amlodipine or metoprolol						
	**Verapamil/Diltiazem**		**Amlodipine**		**Metoprolol**	
**Patients' characteristic**	D vs A	R vs A	D vs A	R vs A	D vs A	R vs A
**Demographic**						
Age, %						
<65	-0.96	-0.96	1.86	0.62	-0.43	-0.97
65-<70	-2.12	-1.7	-0.82	-0.75	-0.26	0.05
70-<75	3.19	1.56	1.97	1.7	0.04	0.07
75-<80	-0.46	-0.07	-3.28	-1.22	-1.59	-1.46
80-<85	1.05	2	-2.4	-3.32	1.44	2.05
≥ 85	1.48	1.72	2.16	3.87	4.1	3.93
Sex, %						
Female	1.46	0.34	0.72	0.37	2.58	3.74
**Medical history, %**						
General						
Diabetes w/o complication	-0.4	-1.41	-2.77	-3.71	-0.19	0.13
Diabetes w/complication	2.04	2.23	-0.43	-1.27	-1.65	-1
Hypertension	-0.66	-1.36	-1.5	-1.32	1.77	2.29
Hypercholesterolemia	1.97	1.25	-3.61	-3.47	-3.84	-4.02
Liver failure						
Mild	1.18	1.4	0.71	0.5	-1.64	-1.01
Moderate or severe	-3.35	-3.35	0	2.54	2.68	0
Cancer						
Solid	0.43	0.77	-0.43	0.12	2.18	2.14
Fluid	1.34	1.45	-0.11	-0.06	0.73	0.03
Metastatic	1.25	0.72	-1.14	-0.6	-0.52	0.91
Obesity	-2.2	-1.86	0.14	-0.69	-0.28	-1.03
Peptic ulcer disease	10.54	10.5	-3.81	-4.4	-2.83	-2.83
Prior hospitalized bleeding event	1.79	2.23	0.08	0.15	2.5	2.75
Smoking	-4.01	-4.24	2.1	1.4	3.99	2.51
Cardiovascular disease						
Acute myocardial infarction						
Past 1-30 days	-1.67	-3.17	1.52	1.43	4.12	2.86
Past 31-180 days	0.53	-0.84	1.22	0.65	-0.05	-0.26
Past 181-365 days	6.43	6.14	-0.71	-1.29	-4.49	-5.46
Coronary revascularization	-0.8	-1.79	2.36	3.82	-1.13	-0.37
Heart failure						
Hospitalized	2.33	1.79	-0.02	0.47	1.62	1.46
Outpatient	2.87	1.2	1.7	0.33	-3.38	-2.1

© 2020 Pham P et al. *JAMA Network Open.*

Coronary artery disease	1.55	0.31	3.43	3.95	0.89	0.22
Other ischemic heart disease	3.06	2.48	3.7	3.72	1.63	0.66
Stroke						
Past 1-30 days	-2.3	-5.26	0.78	1.74	2.43	1.99
Past 31-180 days	-3.59	-4.27	3.14	5.7	-0.98	0.04
Past 181-365 days	4.58	3.46	3.21	2.85	0.74	0.24
Other cerebrovascular disease	-1.37	-1.33	5.01	4.86	0.38	-0.79
Transient ischemic attack (TIA)	-0.22	-0.33	4.95	5.85	1.3	2.09
Cardioversion	2.38	3.2	0.1	-0.36	1.43	0.3
Other medical conditions						
Falls	1.1	0.29	1.76	-0.18	0.67	4.95
Fractures	-0.55	-2.11	-0.07	3.43	-0.67	4.22
Syncope	2.22	2.23	0.18	-0.22	2	3.3
Walker use	-2.3	-1.96	1.32	1.1	0.15	1.28
CCI, Mean (SD)	3.11	2.01	-0.54	-1.12	1.79	3.37
CHA_2DS_2-VASc Score						
0-1	-4.87	-3.9	-0.67	-1.77	0.58	-1.03
2	3.21	2.95	-0.9	-0.63	-1.51	-1.99
3	3.79	3.99	-0.58	0.11	0.48	2.05
≥4	-3.03	-4.97	4.16	4.37	1.22	2.85
HAS-BLED score						
0-1	-0.35	-0.31	2.43	1.72	4.13	2.99
2	-0.16	-0.47	-0.1	-0.96	-3.75	-2.16
3	0.17	0.24	-4.02	-4.06	-2.81	-2.55
≥4	0.3	0.46	1.74	2.94	2.03	1.48
**Medication use, %**						
General						
Estrogen therapy	2.05	2.66	2.84	2.29	0.03	0.03
Histamine 2 antagonists	-2.03	-2.39	3.21	3.33	4.76	1.77
NSAID	-0.06	-1.76	2.28	2.52	-0.69	2.67
Proton pump inhibitors	1.54	2.44	-0.26	0.67	2.04	2.17
SSRI antidepressants	2.8	0.01	-1.32	-0.29	0.26	0.51
Cardiovascular						
ACE inhibitors	-5.15	-4.27	2.78	2.51	1.21	0.78
ARBs	4.64	5.51	0.31	1.07	1.05	2.2
Antiarrhymics	-1.8	-0.79	2.86	3.04	0.39	-1.79
Other anticoagulants	6.58	6.56	0.08	-0.54	-3.16	-4.46
Antiplatelet	-0.05	0.33	3.18	4.62	1.37	0.94
B-blocker	2.92	1.9	2.06	2.21	1.46	1.8
Digoxin	-1.37	-1.35	-0.74	-2.1	1.05	1.91

© 2020 Pham P et al. *JAMA Network Open.*

Diuretics						
Loop	1.28	2.09	0.74	2.21	-0.7	1.68
Potassium sparing	-2.16	-3.42	0.98	0.78	1.87	1.2
Thiazide and other	-1.47	-2.51	2.32	2.9	0.41	0.92
Nitrates	2.95	1.86	2.48	2.64	2.39	0.16
Statins	-0.9	-1.88	-0.19	0.89	0.3	1.01
Fibrates	2.61	5.6	1.46	1.61	3.97	3.05
Other antihyperlipidemic	6.17	6.45	-0.31	0.44	1.01	-0.11
Diabetes related						
Insulin	0.11	1.42	0.6	0.29	1.14	0.54
Metformin	2.65	1.51	-2.99	-3.06	-1.98	-1.07
Sulfonylurea	2.3	2.41	0.13	1.15	-2.95	-3.21
Other	-3.09	-5.7	-3.42	-4.35	-2.66	-1.67
Metabolic inhibitors						
Amiodarone	2.58	3.09	0.12	-0.4	2.65	0.35
Dronedarone	-0.26	-0.01	1.04	1.34	-4.35	-3.56
Azole antifungals	1.48	2.84	2.32	1.38	-3.72	-4.7
Metabolic inducers						
Carbamazepine	6.44	6.23	0.2	1.42	1.2	4.68
Phenytoin	2.62	1.57	0	5.08	0.00	2.67
Phenobarbital	4.16	4.17	3.53	3.53	0.00	0.00
Prescriber specialty						
Internal medicine	-3.37	-2.37	-0.84	-0.40	1.86	-0.05
Family practice	-0.48	-1.99	2.76	3.33	0.21	1.09
Geriatric medicine	0.00	0.00	4.04	4.03	2.68	0.00
Cardiology	-1.96	-1.21	-1.27	-1.93	2.49	0.98
Critical care medicine	0.00	4.72	-0.03	-4.23	3.50	3.49
Hematology	6.23	6.14	3.60	0.00	-3.70	-0.01
Oncology	5.95	6.69	0.00	2.54	0.00	-3.31
**Healthcare utilization**						
Outpatient visits, %						
≤ 9	3.35	2.89	0.23	-0.88	-0.35	-0.21
>9-16	5.5	6.61	-4.75	-4.06	2.84	0.39
>16-29	-6.84	-6.23	0.54	0.4	-2.23	-0.68
>29	-1.39	-2.87	4.97	5.6	-0.36	0.6
Inpatient visits, Mean (SD)	0.01	-1.81	6.92	8.36	4.66	5.33
ER visits	-2.17	-2.9	3.49	2.5	1.43	2.3
Number of Rx, %						
≤ 6	0.24	0.88	1.57	0.43	-3.31	-4.36
>6-9	0.42	-0.95	-2.49	-2.71	-0.29	-0.14
>9-13	-3.58	-2.86	0.72	1.11	-0.06	1.14
>13	2.94	3.04	0.45	1.18	3.6	3.19

© 2020 Pham P et al. *JAMA Network Open.*

V/D: verapamil/diltiazem; A: amlodipine;
CCI: Charlson comorbidity index; NSAID: nonsteroidal anti-inflammatory drugs; SSRI: selective serotonin reuptake inhibitors; ACE inhibitors: Angiotensin-converting enzyme inhibitors; ARB: Angiotensin II receptor blockers; ER: emergency room

© 2020 Pham P et al. *JAMA Network Open.*

**eTable 7: Number and incident rate of adverse bleeding events among concomitant users of DOACs with verapamil/diltiazem and with amlodipine**									
**Bleeding categories**	**Dabigatran**			**Rivaroxaban**			**Apixaban**		
	**Event (N)**	**Crude ER**	**Adj ER**	**Event (N)**	**Crude ER**	**Adj ER**	**Event (N)**	**Crude ER**	**Adj ER**
Verapamil/Diltiazem									
Overall bleed	51	256.2	244.9	56	274.8	270.5	17	159.1	165.3
Overall GI bleed	30	146.0	149.7	23	108.0	84.0	6	54.8	50.0
Major/moderate bleed	12	56.9	54.4	15	69.4	73.0	4	36.5	32.5
Major/moderate GI bleed	7	33.2	29.6	5	23.0	19.0	1	9.1	9.9
Minor bleed	49	245.3	239.4	52	254.4	246.7	15	140.2	152.9
GI minor bleed	29	141.3	145.6	22	103.3	77.4	6	54.8	50.0
Stroke + SE	0	NA	NA	1	4.4	8.8	0	NA	NA
Amlodipine									
Overall bleed	71	177.0	158.4	98	270.1	273.4	34	207.7	185.1
Overall GI bleed	32	77.7	68.6	47	125.9	131.4	14	82.1	71.9
Major/moderate bleed	12	28.8	24.1	22	58.0	59.9	4	23.0	20.8
Major/moderate GI bleed	7	16.8	13.1	12	31.4	37.2	1	5.8	4.7
Minor bleed	68	169.4	151.1	93	255.5	258.4	32	194.9	174.5
GI minor bleed	31	75.6	66.8	44	117.5	123.7	14	82.1	71.9
Stroke + SE	0	NA	NA	1	2.6	2.2	1	5.5	12.8
Abbreviations: ER=event rate; Adj: adjusted; GI=gastrointestinal; SE: systemic embolism; D=dabigatran; R=rivaroxaban; A=apixaban; DOAC=direct acting oral anticoagulants. *rate per 1000 person years									

© 2020 Pham P et al. *JAMA Network Open.*

**eTable 8: Number and incident rate of adverse bleeding event among concomitant users of DOACs with verapamil/diltiazem and with metoprolol**									
**Bleeding categories**	**Dabigatran**			**Rivaroxaban**			**Apixaban**		
	**Event (N)**	**Crude ER**	**Adj ER**	**Event (N)**	**Crude ER**	**Adj ER**	**Event (N)**	**Crude ER**	**Adj ER**
Verapamil/Diltiazem									
Overall bleed	53	248.2	291.3	62	278.5	234.7	19	163.9	170.5
Overall GI bleed	30	139.1	171.6	25	108.0	84.7	7	62.1	88.7
Major/moderate bleed	12	54.4	85.0	15	66.4	55.8	5	44.2	29.6
Major/moderate GI bleed	7	31.8	50.0	5	21.9	18.6	1	8.8	4.7
Minor bleed	51	237.6	275.6	58	258.4	223.4	17	145.3	140.2
GI minor bleed	29	134.3	167.5	24	103.3	82.1	7	62.1	88.7
Stroke +SE	0	NA	NA	1	4.4	3.3	0	NA	NA
Metoprolol									
Overall bleed	80	201.1	199.7	127	316.8	314.6	45	223.4	219.4
Overall GI bleed	32	76.7	72.3	53	122.6	119.0	19	94.5	104.0
Major/moderate bleed	11	24.5	25.2	23	55.8	57.3	4	15.3	20.1
Major/moderate GI bleed	5	9.9	9.1	11	26.3	26.3	2	5.1	11.0
Minor bleed	78	196.0	194.9	122	302.6	300.4	42	211.7	209.5
GI minor bleed	30	74.1	70.4	51	117.5	114.2	18	94.5	104.0
Stroke + SE	1	2.2	2.6	1	2.2	2.2	0	NA	NA
Abbreviations: ER=event rate; Adj: adjusted; GI=gastrointestinal; SE: systemic embolism; D=dabigatran; R=rivaroxaban; A=apixaban; DOAC=direct acting oral anticoagulants.<br>*rate per 1000 person years									

© 2020 Pham P et al. *JAMA Network Open.*

eTable 9: Hazard ratio of adverse bleed events among concomitant users of direct oral anticoagulant and verapamil/diltiazem versus amlodipine			
**Bleeding categories**	**Dabigatran HR (95% CI)**	**Rivaroxaban HR (95% CI)**	**Apixaban HR (95% CI)**
**Unadjusted analysis**			
Overall bleed	1.40 (0.98-2.01)	1.02 (0.74-1.42)	0.77 (0.43-1.37)
Overall GI bleed	1.84 (1.12-3.03) *	0.85 (0.52-1.41)	0.67 (0.26-1.75)
Major/moderate bleed	1.95 (0.87-4.34)	1.22 (0.63-2.36)	1.60 (0.40-6.41)
Major/moderate GI bleed	1.97 (0.69-5.64)	0.73 (0.26-2.08)	1.79 (0.11-28.69)
Minor bleed	1.40 (0.97-2.03)	1.00 (0.71-1.41)	0.72 (0.39-1.32)
GI minor bleed	1.84 (1.11-3.06) *	0.87 (0.52-1.46)	0.67 (0.26-1.75)
**Diltiazem alone**			
Overall bleed	1.56 (1.06-2.30) *	1.06 (0.75-1.51)	1.11 (0.60-2.04)
Overall GI bleed	2.06 (1.20-3.54) *	0.58 (0.32-1.08)	0.81 (0.28-2.37)
Major/moderate bleed	2.30 (0.95-5.55)	1.42 (0.73-2.76)	2.02 (0.44-9.17)
Major/moderate GI bleed	2.17 (0.65-7.27)	0.64 (0.21-1.93)	2.80 (0.14-55.74)
Minor bleed	1.61 (1.09-2.39) *	1.01 (0.70-1.46)	1.08 (0.57-2.03)
GI minor bleed	2.12 (1.23-3.64) *	0.55 (0.29-1.05)	0.81 (0.28-2.37)
**Verapamil alone**			
Overall bleed	1.34 (0.64-1.82)	0.71 (0.35-1.44)	0.13 (0.01-1.94)
Overall GI bleed	2.70 (1.15-6.34) *	0.83 (0.32-2.13)	0.33 (0.02-5.18)
Major/moderate bleed	2.12 (0.46-9.74)	0.51 (0.09 -2.93)	NA
Major/moderate GI bleed	2.79 (0.43-17.98)	NA	NA
Minor bleed	1.29 (0.60-2.80)	0.74 (0.36-1.51)	0.14 (0.01-2.08)
GI minor bleed	2.44 (0.99-6.01)	0.87 (0.34-2.25)	0.33 (0.02-5.18)
**15-day gap**			
Overall bleed	1.35 (0.97-1.88)	0.85 (0.62-1.17)	1.02 (0.58-1.81)
Overall GI bleed	1.87 (1.19-2.93) *	0.60 (0.35-1.00)	0.71 (0.25-2.01)
Major/moderate bleed	2.52 (1.18- 5.41) *	1.18 (0.64-2.18)	2.41 (0.60-9.64)
Major/moderate GI bleed	3.58 (1.25-10.21) *	0.64 (0.24-1.68)	2.17 (0.11-43.09)
Minor bleed	1.36 (0.98-1.90)	0.84 (0.60-1.16)	0.98 (0.54-1.77)
GI minor bleed	1.83 (1.16 -2.90) *	0.57 (0.34-0.98) *	0.71 (0.25-2.01)
**Any diagnosis code position for outcome**			
Overall bleed	1.74 (1.49-2.02)	0.87 (0.77-0.99) †	1.01 (0.81-1.27)
Overall GI bleed	1.93 (1.55-2.41) †	0.46 (0.36-0.57) †	0.45 (0.29-0.71) †
Major/moderate bleed	1.89 (1.07-3.32) †	1.06 (0.74-1.55)	1.92 (0.71-5.16)
Major/moderate GI bleed	1.97 (0.75-5.17)	0.30 (0.15-0.58) †	5.66 (0.42-75.69)
Minor bleed	1.75 (1.50-2.05) *	0.87 (0.76-0.99) †	1.02 (0.81-1.29)
GI minor bleed	1.91 (1.53-2.39) †	0.43 (0.34-0.54) †	0.41 (0.25-0.65) †
**Standardized mortality ratio weight**			
Overall bleed	1.31 (0.97-1.77)	1.45 (0.86-1.53)	0.48 (0.13-1.80)
Overall GI bleed	2.16 (1.43-3.26) *	1.19 (0.76-1.87)	0.56 (0.24-1.30)
Major/moderate bleed	2.34 (1.21-4.52) *	1.64 (0.92-2.90)	1.23 (0.41-3.69)
Major/moderate GI bleed	2.60 (1.08-6.24) *	1.15 (0.44-3.00)	1.03 (0.12-8.78)
Minor bleed	1.32 (0.97-1.79)	1.11 (0.82-1.49)	0.60 (0.35-1.03)
GI minor bleed	2.29 (1.50-3.49) *	1.20 (0.75-1.89)	0.56 (0.24-1.30)

© 2020 Pham P et al. *JAMA Network Open.*

Abbreviations: HR=hazard ratio; CI=confidence interval; GI=gastrointestinal; D=dabigatran; R=rivaroxaban; A=apixaban; DOAC=direct acting oral anticoagulants
NA: no event in one of the comparison groups
*Denotes statistically significant results at *P*<0.05
†Denotes proportional hazard assumption violated as the interaction between treatment and time was significant

© 2020 Pham P et al. *JAMA Network Open.*

eTable 10: Hazard ratio of adverse bleed events among concomitant use of direct oral anticoagulant and verapamil/diltiazem versus metoprolol			
**Bleeding categories**	**Dabigatran HR (95% CI)**	**Rivaroxaban HR (95% CI)**	**Apixaban HR (95% CI)**
**Unadjusted result**			
Overall bleed	1.21 (0.85-1.72)	0.89 (0.65-1.21)	0.73 (0.42-1.28)
Overall GI bleed	1.78 (1.08-2.95) *	0.88 (0.54-1.44)	0.67 (0.28-1.59)
Major/moderate bleed	2.21 (0.95-5.11)	1..21 (0.63-2.33)	2.87 (0.68-12.02)
Major/moderate GI bleed	3.19 (0.93-10.91)	0.84 (0.29-2.43)	1.75 (0.11-28.04)
Minor bleed	1.19 (0.83-1.70)	0.86 (0.63-1.19)	0.68 (0.38-1.23)
GI minor bleed	1.78 (1.07-2.97) *	0.88 (0.53-1.45)	0.67 (0.28-1.59)
**Diltiazem alone**			
Overall bleed	1.35 (0.93-1.95)	0.77 (0.54-1.10)	0.72 (0.39-1.34)
Overall GI bleed	2.17 (1.28-3.68) *	0.76 (0.43-1.34)	0.52 (0.19-1.44)
Major/moderate bleed	2.67 (1.15-6.20) *	1.07 (0.52-2.20)	1.86 (0.42-8.15)
Major/moderate GI bleed	4.31 (1.20-15.49) *	0.91 (0.29-2.80)	0.54 (0.03-11.09)
Minor bleed	1.36 (0.94-1.97)	0.75 (0.52-1.09)	0.57 (0.28-1.13)
GI minor bleed	2.23 (1.31-3.78) *	0.76 (0.43-1.36)	0.52 (0.19-1.44)
**Verapamil alone**			
Overall bleed	1.76 (1.01-3.06) *	0.72 (0.37-1.40)	1.00 (0.40-2.52)
Overall GI bleed	2.92 (1.40-6.11) *	0.52 (0.15-1.78)	2.06 (0.77-5.48)
Major/moderate bleed	5.84 (2.20-15.49) *	0.71 (0.15-3.26)	NA
Major/moderate GI bleed	9.82 (1.40-40.10) *	NA	NA
Minor bleed	1.49 (0.82-2.72)	0.75 (0.39-1.47)	1.04 (0.41-2.62)
GI minor bleed	2.71 (1.26-5.87) *	0.54 (0.16-1.85)	2.06 (0.77-5.48)
**15-day gap**			
Overall bleed	1.35 (0.98-1.86)	0.70 (0.51-0.96) *	0.83 (0.49-1.38)
Overall GI bleed	2.01 (1.26- 3.20) *	0.60 (0.36-0.99) *	0.77 (0.36-1.65)
Major/moderate bleed	2.71 (1.36- 5.38) *	0.96 (0.50-1.83)	2.09 (0.54-8.06)
Major/moderate GI bleed	4.75 (1.69-13.39) *	0.92 (0.33-2.51)	0.41 (0.02-8.48)
Minor bleed	1.31 (0.95-1.81)	0.70 (0.51-0.96) *	0.69 (0.40-1.21)
GI minor bleed	2.01 (1.25-3.22) *	0.60 (0.36-1.00)	0.77 (0.36-1.65)
**Any diagnosis code position for outcome**			
Overall bleed	1.23 (1.07-1.40) †	0.91 (0.81-1.03) †	0.67 (0.52-0.87) †
Overall GI bleed	1.92 (1.56-2.37) †	0.63 (0.51-0.79) †	0.35 (0.23-0.55) *
Major/moderate bleed	1.72 (1.06-2.77) *	0.88 (0.59-1.31)	1.71 (0.73-4.00)
Major/moderate GI bleed	3.78 (1.64-8.72) *	0.36 (0.16-0.82) †	0.26 (0.03-2.14)
Minor bleed	1.21 (1.06-1.38) †	0.91 (0.80-1.03) †	0.64 (0.49-0.84) †
GI minor bleed	1.90 (1.54-2.35) †	0.62 (0.49-0.78) †	0.35 (0.22-0.55) *
**Standardized mortality ratio weight**			
Overall bleed	1.26 (0.93-1.72)	0.87 (0.66-1.15)	0.86 (0.52-1.43)
Overall GI bleed	2.01 (1.31-3.09) *	0.80 (0.52-1.22)	1.09 (0.48-2.50)
Major/moderate bleed	3.69 (1.72-7.91) *	1.26 (0.72-2.21)	10.77 (2.12-54.73) *
Major/moderate GI bleed	3.27 (1.24-8.63) *	0.72 (0.28-1.84)	10.28 (0.29-355.88)

© 2020 Pham P et al. *JAMA Network Open.*

Minor bleed	1.27 (0.92-1.73)	0.85 (0.64-1.13)	0.78 (0.46-1.33)
GI minor bleed	2.12 (1.36-3.29) *	0.79 (0.51-1.23)	1.09 (0.48-2.50)

Abbreviations: HR=hazard ratio; CI=confidence interval; GI=gastrointestinal; D=dabigatran; R=rivaroxaban; A=apixaban; DOAC=direct acting oral anticoagulants

NA: no event in one of the comparation groups

*Denotes statistically significant results at $P<0.05$

†Denotes proportional hazard assumption violated as the interaction between treatment and time was significant and a covariate for time was entered into the model.

© 2020 Pham P et al. *JAMA Network Open.*
